# Biological properties and potential of compounds extracted from red seaweeds

**DOI:** 10.1007/s11101-022-09826-z

**Published:** 2022-07-01

**Authors:** M. Carpena, P. Garcia-Perez, P. Garcia-Oliveira, F. Chamorro, Paz Otero, C. Lourenço-Lopes, Hui Cao, J. Simal-Gandara, M. A. Prieto

**Affiliations:** 1 Nutrition and Bromatology Group, Department of Analytical Chemistry and Food Science, Faculty of Science, Universidade de Vigo, E-32004 Ourense, Spain; 2 Centro de Investigação de Montanha (CIMO), Instituto Politécnico de Bragança, Campus de Santa Apolonia, 5300-253 Bragança, Portugal

**Keywords:** Red algae, Sulfated polysaccharides, Bioactive compounds, Biological properties, Extraction techniques

## Abstract

Macroalgae have been recently used for different applications in the food, cosmetic and pharmaceutical industry since they do not compete for land and freshwater against other resources. Moreover, they have been highlighted as a potential source of bioactive compounds. Red algae (Rhodophyta) are the largest group of seaweeds, including around 6000 different species, thus it can be hypothesized that they are a potential source of bioactive compounds. Sulfated polysaccharides, mainly agar and carrageenans, are the most relevant and exploited compounds of red algae. Other potential molecules are essential fatty acids, phycobiliproteins, vitamins, minerals, and other secondary metabolites. All these compounds have been demonstrated to exert several biological activities, among which antioxidant, anti-inflammatory, antitumor, and antimicrobial properties can be highlighted. Nevertheless, these properties need to be further tested on in vivo experiments and go in-depth in the study of the mechanism of action of the specific molecules and the understanding of the structure–activity relation. At last, the extraction technologies are essential for the correct isolation of the molecules, in a cost-effective way, to facilitate the scale-up of the processes and their further application by the industry. This manuscript is aimed at describing the fundamental composition of red algae and their most studied biological properties to pave the way to the utilization of this underused resource.

## Introduction

Marine biotechnology (also called blue biotechnology) consists of the application of biological resources from the sea for industrial, medical or environmental purposes (Thompson et al. 2017), which constitutes a precious economical sector presenting a yearly turnover of 3.93 $ together with great expectations, including many different subjects and organisms which could report benefits to the industry (Bloch and Tardieu-Guigues 2014; Thompson et al. 2017). Among them, micro- and macro-algae have gained much attention as natural sources of bioactive compounds that exhibit a great applicability as dietary ingredients and other industrial processes (Bloch and Tardieu-Guigues 2014; Sudhakar et al. 2018). On these bases, seaweeds have been consumed for many years in Asian countries, since they constitute a rich source of fiber, vitamins, minerals and antioxidants, thus prompting an increase in their consumption and, consequently, promoting intense efforts on the characterization of their health-enhancing properties (Cardozo et al. 2007; Gómez-Ordóñez et al. 2012; Cian et al. 2015; Rudtanatip et al. 2018; Gurpilhares et al. 2019).

Macroalgae are becoming of great importance within the aquaculture industry, as they are a potential feeding for marine organisms, including corals (Gurgel and Lopez-Bautista 2007), and they do not compete against other resources proceeding from the land and freshwater. Moreover, macroalgae are remarkable for their rapid growth rate and high polysaccharides content, becoming great candidates for biofuel production (Sudhakar et al. 2018). In addition, their associated positive effects on health and biological activities must be highlighted as of great importance on food, pharmaceutical and cosmetic fields (Gurpilhares et al. 2019). Concerning their classification, marine macroalgae are classified into three groups, according to their main pigments as green (Chlorophyta), red (Rhodophyta) and brown algae (Phaeophyta) (Mohamed et al. 2012; Belghit et al. 2017; Davies et al. 2019). Regarding their chemical composition, macroalgae exhibit a high content of water, carbohydrates and proteins and a low lipid percentage (Sudhakar et al. 2018). Considering their differential composition, the phylum Rhodophyta presents the highest proportion of bioactive compounds, accounting for more than 1600 individual compounds, representing the 53% of bioactive compounds reported in algae (Leal et al. 2013).

**Fig. 1:**
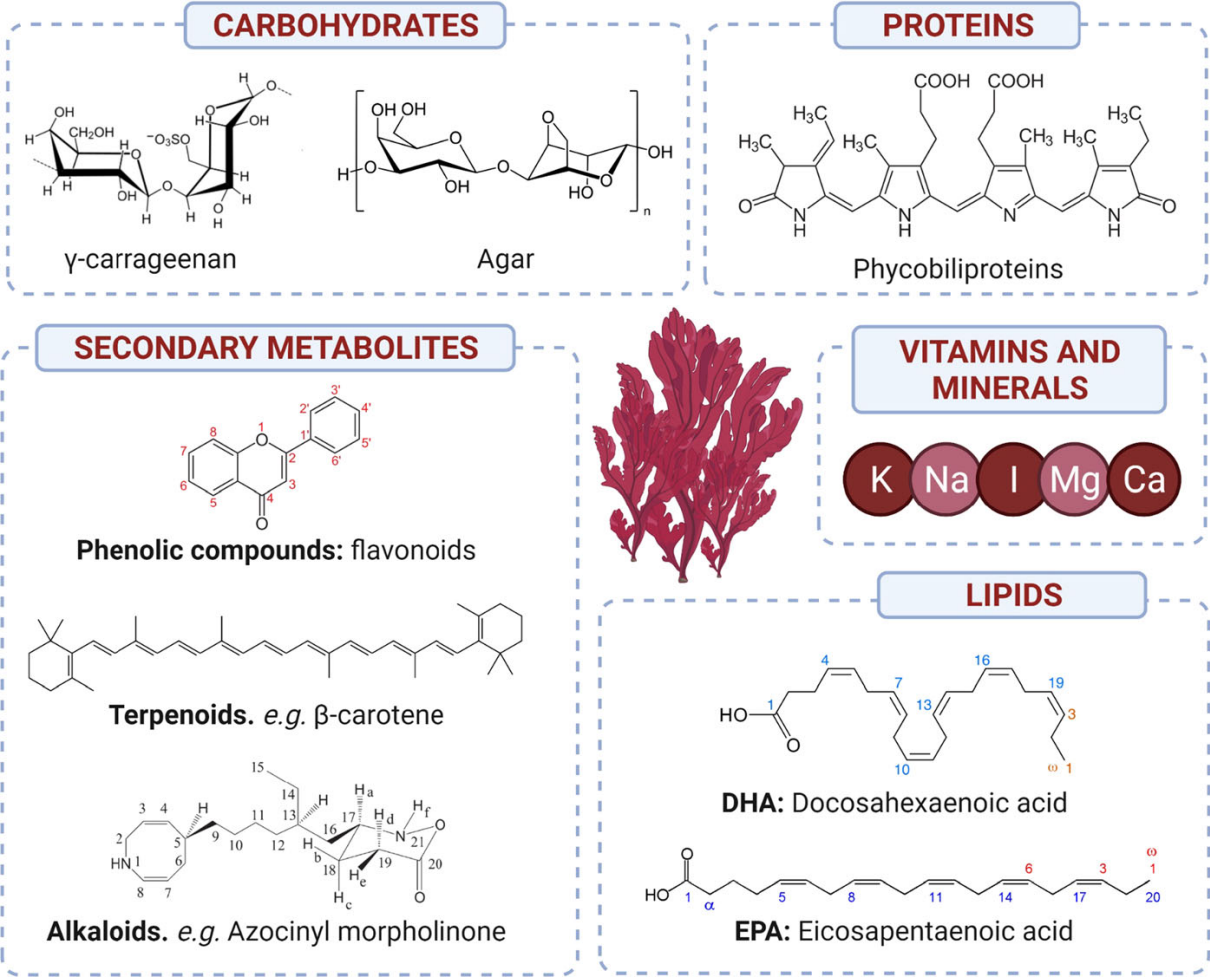
Main bioactive compounds of red algae

Additionally, red algae form the largest group of seaweeds, including around 6,000 different species. With respect of biological aspects, red seaweeds are smaller than green and brown algae, being usually found in equatorial regions along intertidal areas and beyond. Due to their color-based classification, red algae contain a specific combination of pigments, *i.e.*: chlorophyll *a* and *d*, carotenoids and phycobiliproteins (Gurgel and Lopez-Bautista 2007; Cian et al. 2015). Considering their nutritional composition, red algae have been proposed to be incorporated to the human diet because they present the highest levels of proteins among algae, and huge amounts of carbohydrates and minerals (Belghit et al. 2017; Øverland et al. 2019; Torres et al. 2019), as depicted in Fig. 1. In particular, the families *Gelidiaceae* and *Gracilariaceae* have been revealed as economically interesting, as they are the major sources of agar and carrageenans, reaching worldwide production yields of 10,000 tons and 25,000 tons, respectively, valued at 200 $ million each (Cardozo et al. 2007)*.* Consequently, the cultivation of red seaweeds is mainly aimed at the production of carrageenans, traditionally extracted from *Chondrus crispus* wild populations in Canada, Ireland, Portugal, Spain and France and from *Gigartina* collected in South America and Southern Europe. However, the growing demands of carrageenans motivated the establishment of macroalgae farming systems with *Euchema* sp. in Philippines (Valderrama et al. 2013; Hedberg et al. 2018), becoming the major producer worldwide and spreading macroalgae cultivation along other Asian countries, promoting the production of *Porphyra* sp. (nori), *Kappaphycus alvarezii* and *Eucheuma denticulatum* (Valderrama et al. 2013). According to the Food and Agriculture Organization (FAO), in the last decade red macroalgae production reached almost 9 million wet tons, representing the 47% of the total production of cultivated seaweeds (Valderrama et al. 2013).

On these bases, due to the positive nutritional and economic impact attributed to red algae, greater efforts are required to promote their exploitation and diffusion along Western countries. Thus, this review is aimed at describing the chemical composition of red algae, with a special focus on compounds with health-enhancing properties. Consequently, a deep description of the biological properties associated with red algae extracts is provided, focusing on their antioxidant, antimicrobial, anticancer, anti-inflammatory, antidiabetic, and metabolic regulator activities. Furthermore, a detailed insight on the extraction methodologies applied to the isolation and production of bioactive compounds is also described, with the aim of providing evidence on the beneficial properties of these marine organisms to be incorporated into different food, cosmetic, and pharmaceutical formulations.

## Chemical composition of red algae

Marine algae are great candidates for being included in dietary regimes due to their nutritional and chemical composition. Over 3000 marine natural products extracted from them have been largely identified (Leal et al. 2013; Belghit et al. 2017). However, seaweeds have not been commonly exploited with pharmaceutical and/or nutraceutical purposes, but a growing interest, led by the traditional consumption in Asian countries, has promoted the research of chemical constituents from algae (Sangha et al. 2013). Consequently, a structural and functional characterization of red seaweed constituents, which are responsible for the biological properties associated with these organisms, should be performed to assess their incorporation into the diet. Taken all together, the chemical composition of red seaweeds is composed of carbohydrates, lipids, proteins, peptides, vitamins, minerals and secondary metabolites (Barbalace et al. 2019; Torres et al. 2019). Figure 1 summarized the most relevant constituents found in red seaweeds. In Table 1, main applications of these constituents have been compiled.

**Table 1:** Applications related to the main compounds found in red algae

Compound	Application	Reference
Carrageenan	Jellifying, stabilizing and emulsifying properties	Cunha and Grenha (2016)
	Antioxidant activity	Silva et al. (2012), Gómez-Ordóñez et al. (2014), Cian et al. (2015)
	Antithrombotic, anti-inflammatory and antidiabetic activities	Holdt and Kraan (2011), Gómez-Ordóñez et al. (2012), Cian et al. (2015)
	Oil binding properties and emulsifier	Suleria et al. (2016)
	Antitumor, antiviral, anticoagulant and immunomodulation activities	Cardozo et al. (2007), Gómez-Ordóñez et al. (2012), Mohamed et al. (2012), Silva et al. (2012), Cunha and Grenha (2016), Davies et al. (2019), Torres et al. (2019)
	Cholesterol and lipid-lowering effects	Mohamed et al. (2012)
	Serum cholesterol and triglyceride levels reduction	Silva et al. (2012)
Agar	Gelling and stabilizing properties	Davies et al. (2019)
	Texture improvement and stabilizing properties	Suleria et al. (2016)
	Viscosifying and emulsifying properties Anticoagulant	Maciel et al. (2016)
	Antitumor, anti-aggregation, antioxidant, UV rays’ absorption	Holdt and Kraan (2011)
Polar lipids, PUFAs and sulfolipids	Anti-inflammatory, immunomodulatory, anti-angiogenic, and neuroprotective, antimicrobial, antifungal properties	Maciel et al. (2016), Belghit et al. (2017), Gurpilhares et al. (2019)
	Reducing coronary diseases, diabetes, and osteoarthritis	Mohamed et al. (2012)
Lectins	Carcinoma inhibition. Anti-HIV, anti-influenza, anti-coronavirus, anti-hepatitis, anti- herpes simplex virus, miscellaneous, anti-cancer, anti-nociceptive, anti-inflammatory, anti-microbial, anti-encephalitis	Holdt and Kraan (2011), Singh and Walia (2018)
Phycobiliprotein	Natural food colorant	Suleria et al. (2016)
	Fluorescent pigments: medical reagents	Sudhakar et al. (2018)
	Antioxidant properties, prevention of neurodegenerative diseases, cancer and gastric ulcers	Mohamed et al. (2012)
	Anti-inflammatory, antioxidant, antiviral, antitumor, serum lipid reducing, neuroprotective, hypercholesterolemic, liver protecting, hepatoprotective	Holdt and Kraan (2011)
Sulfated polysaccharides	Immune stimulant effect	Rudtanatip et al. (2018)
	LDL cholesterol reduction and HDL increase	Mohamed et al. (2012)
Porphyran	Anti-allergic activity, scavenging free radical activity, antitumor activities	Mohamed et al. (2012), Davies et al. (2019), Øverland et al. (2019)
	Elevation of primary antibody response, macrophages stimulation and Th-2 type immune system suppression without affecting Th-1 type immune system	Mohamed et al. (2012)
	Anticoagulant, anti-hypercholesterolemic, antitumor	Holdt and Kraan (2011)
	Hepatoprotective properties	Mohamed et al. (2012)
Fatty acids	Antifungal activity	De Corato et al. (2017)
Pigments and MAA	Photo-protective compounds. Antioxidant properties	Cardozo et al. (2007), Lalegerie et al. (2019)
	Anticancer, anti-proliferative and antitumor effects	Mohamed et al. (2012)
Phloroglucinol	Anti-allergic, antifungal, antimicrobial and anti-feeding	Gómez-Ordóñez et al. (2012)
Soluble dietary fiber	Retard digestion and glucose absorption	Mohamed et al. (2012)
	Prebiotic	Cian et al. (2015)
Iodine	Antioxidant, anti-goiter and anticancer	Mohamed et al. (2012)
Glycolipids	Antimicrobial, antifungal, antitumor, antiviral, anti-inflammatory activities	Maciel et al. (2016)

### Carbohydrates

Although monosaccharides have been reported in red algae, little attention has been paid to these molecules, being poorly characterized. In this sense, several free sugars have been found in red algae including fucose, xylose, mannose, galactose, and glucose (Gómez-Ordóñez et al. 2010, 2014). On the contrary, polysaccharides constitute the major constituents in marine algae, including the red ones, which enables the enhancement of the commercial value of red algae, thanks to their potential applications in the food industry, where they are usually exploited as an efficient source of dietary fiber, but also in both the pharmaceutical and biomedical industries. According to their prevalence in algal sources, agar and carrageenan, both sulfated polysaccharides known as phycocollooids, are the most relevant carbohydrates in red seaweeds, accounting for up to 40–50% of the dry weight (Torres et al. 2019), followed by other polysaccharides found in significantly lower amounts, such as xylans, sulphated galactans and porphyrans (Øverland et al. 2019).

Thus, carrageenan has been reported as the major representative of red marine algae, representing the most relevant constituent of algal cell walls. This polysaccharide is a sulfated polygalactan mainly formed by α- and β-D-galactopyranose subunits linked by two different types of glycosidic bonds: α (1 → 3) and β (1 → 4). According to the configuration and proportion of such bonds, different kind of carrageenans have been identified, accounting for more than 15 types with industrial relevance currently described (Prado-Fernández et al. 2003; Hilliou et al. 2006; Cunha and Grenha 2016), being divided into three groups, as a general rule: kappa, iota and lambda, κ, ι, λ carrageenans, respectively. Such carrageenan classification mostly owes to structural purposes and the heterogeneous existence of chemical substitutions, which lead to specific physicochemical properties that contribute to the differential features and applicability associated with their derivative products (Cunha and Grenha 2016). Furthermore, specific distributions of carrageenans have been attributed to individual algal species. For instance, *C. crispus* presents a mixture of both κ- and λ-carrageenans that cannot be separated during their large-scale extraction procedure. Indeed, for the production of individual compounds, different algal sources are employed, since κ-carrageenan is usually extracted from *Kappaphycus alvarezii*, whereas λ- carrageenan is isolated from different species from the genus *Gigartina* (Cunha and Grenha 2016; Torres et al. 2019). Considering its food application, carrageenans have been identified as Generally Recognized As Safe (GRAS), so they have been collectively approved for their use on human consumption. Due to their chemical nature as complex polysaccharides, carrageenans cannot be digested by human digestive tract, although they can be fermented by the colonic microbiota (Gómez-Ordóñez et al. 2012). On these bases, to date, these substances are not known for their potential to be added to human diet (Necas and Bartosikova 2013; Torres et al. 2019). Instead, carrageenans are well-known for their additional properties that guide their industrial applications as gelling, stabilizing and emulsifying agents (Cunha and Grenha 2016; Sudhakar et al. 2018). Besides such food-related properties, a number of reports have also listed several bioactivities attributed to carrageenans, including anticoagulant, antiviral, antioxidant and antitumoral effects, together with immunomodulatory and cholesterol-lowering properties (Pangestuti and Kim 2014; Cunha and Grenha 2016).

In addition to carrageenans, agar constitutes another relevant polysaccharide from algae. Concerning its chemical structure, agar is a type of phycocolloid belonging to galactan family composed by α (1 → 4)-3,6-anhydro-L-galactose and β (1 → 3)-D-galactose residues, accompanied by a slight sulfate content. The agar proportions with respect to total algal weight vary among the species and its abundance and quality are also highly dependent of environmental factors and seasonal variations, together with the physicochemical composition of each alga (Cardozo et al. 2007). With respect to its applicability, agar has been identified as GRAS, being already assessed as a safe additive to be incorporated into different food matrices. Thus, regarding its possible incorporation to diet, and keeping in mind its polysaccharidic nature, it cannot be digested by the human gastrointestinal tract, as it occurred with carrageenans, although it can metabolized by intestinal bacteria to give rise to D-galactose (Sudhakar et al. 2018). Hence, both polysaccharides have been suggested to promote prebiotic effects, improving the performance of human digestion (Mohamed et al. 2012; Cian et al. 2015). Concerning its physicochemical properties as food additives, agar is mainly used as a gelling and stabilizing agent, as currently found for many food matrices, but it has also been exploited as cryoprotectants and solidifying agents, incorporated as ingredients of growth media for the in vitro culture of different organisms, including plants and microorganisms (Sudhakar et al. 2018; Torres et al. 2019).

### Lipids

In general, marine algae present a low content of lipids, which ranges between 1 and 5% of total dry weight. However, they do possess a high proportion of poly-unsaturated fatty acids (PUFAs) (Belghit et al. 2017; Praveen et al. 2019) and other lipids like sterols but also make part of different heterogeneous compounds, such as glycolipids and phospholipids (Torres et al. 2019). Among fatty acids, marine algae are rich in essential fatty acids, especially omega-3 fatty acids (ω-3). In particular, red macroalgae contain C-20 ω-3 PUFAs, including eicosapentaenoic acid, α-linolenic acid, and docosahexaenoic acid (Maciel et al. 2016; Torres et al. 2019). Furthermore, besides ω-3 PUFAs, omega-6 fatty acids (ω-6) have been found in red algae in a much lesser extent, being mostly represented by arachidonic acid. As a result, they show a very low ω-6/ω-3 rate, suggesting a healthy lipid profile in which the prevalence of ω-3 PUFAs over ω-6 indicates an efficient profile with beneficial properties on the prevention of cardiovascular diseases, osteoarthritis and diabetes, together with enhanced anti-inflammatory and anti-thrombotic properties (Macartain et al. 2007; Maciel et al. 2016). Moreover, besides such bioactivities, additional biological properties have been associated with those essential fatty acids derived from marine sources, including red algae, acting as antifungal, antibacterial, antiviral and antitumor agents (Pereira 2011; Torres et al. 2019).

### Proteins and peptides

Among the different algae classes found in marine ecosystems, red seaweeds exhibit the highest content of proteins, followed by green and, finally, brown algae (Belghit et al. 2017; Øverland et al. 2019). As a general rule, the protein content of algae usually ranges between 5 and 20%, although red algae may achieve greater proportions, with maximum values reaching 47% of total dry weight (Cian et al. 2015; Rudtanatip et al. 2018; Praveen et al. 2019). Nevertheless, proteins depict a species-dependent occurrence, considering that some species, such as those from *Gracilaria* genus present a low protein content below 5%, whereas others like *Pyropia tenera* shows a protein content of 37% of dry weight. Moreover, it should be noted that protein content also shows a significant influence on several experimental, environmental and geographical factors, such as the extraction and purification procedures, seasonal variations and the collection area (Holdt and Kraan 2011).

Concerning the amino acidic composition of red algae proteins, a high content of essential amino acids has been reported, being aspartic acid and glutamic acid the most prevalent residues, accounting for up to 22–44% of total amino acids making part of red algae proteins (Cian et al. 2015). Thus, such elevated proportion of acidic amino acids has been identified as a specific trait of red seaweeds, being responsible for their organoleptic characteristics, such as flavor and taste (Cian et al. 2015). Moreover, with respect of total proteins, phycobiliproteins constitute the most prevalent proteins in red seaweeds achieving values up to 50% of total protein content, and causing the reddish coloration attributed to these species (Niu et al. 2007). Among phycobiliproteins, phycoerythrin and phycocyanin, together with their combination, have been reported as the major constituents of this family of biomolecules (Cian et al. 2015). Deriving from proteins, bioactive peptides have been isolated in different red algal sources, mostly in *Palmaria* spp. and *Porphyra* spp., although a limited application of these molecules was observed, being only exploited as food additives in a number of functional foods commercialized in Asian countries (Lafarga et al. 2020). In a lesser extent, lectins have been also identified as versatile proteins widely distributed in red algae, acting as cell signaling mediators and antimicrobial compounds (Liao et al. 2003).

### Vitamins and minerals

In addition to the above-mentioned macronutrients associated with red seaweeds, these organisms also contain several nutrients found in very scarce concentrations but developing a significant beneficial effect on human health. Among these nutrients, vitamins play a major role, as great variety of these compounds have been isolated from red algae, including both water-soluble vitamins, B1, B2, B12, and C) and lipid-soluble vitamins, such as pro-vitamin A (β-carotene) and vitamin E (Škrovánková 2011). Indeed, vitamins from marine sources have been already used for the enrichment of functional foods (Figure 1).

Besides such organic micronutrients, minerals have been also reported in red seaweeds as inorganic micronutrients. Due to their marine habitats, red algae are able to accumulate great mineral concentrations, proceeding from seawater (Rosemary et al. 2019). In this sense, Na, K, Ca, and Mg, have been identified in high concentrations, ranging 0.4–4 g per 100 g of red seaweeds, such as *Chondrus* spp. and Nori, whereas trace elements, such as Fe, Zn, Mn, and Cu have been reported, as well, in concentrations up to 10 mg per 100 g (Rupérez 2002). Moreover, special attention has been paid to iodine, since this essential mineral has been also reported in significant amounts in *Gracilaria lemaeniformis*, contributing to the promotion of thyroid function (Wen et al. 2006).

### Secondary metabolites

Besides the previously described compounds from red algae, as part of their primary metabolism, these seaweeds also biosynthesize different compounds with associated biological activities as a result of their secondary metabolism, committed to the development of defensive and adaptative responses against environmental stresses. In the particular case of red seaweeds, multiple reports have indicated the presence of phenolic compounds, terpenoids, and alkaloids as the most prevalent secondary metabolites (Aziz et al. 2020).

Among phenolic compounds, ubiquitously found natural phenolics, mostly phenolic acids and flavonoids are present in red seaweeds, together with other phenolic compounds characteristic of marine sources, such as phlorotannins and bromophenols, all characterized by their potent antioxidant associated activity. Thus, phenolic acids reported in red algae are *p*-coumaric acid, caffeic acid, salicylic acid, hypogallic acid, and chlorogenic acid (Kazłowska et al. 2010; Onofrejová et al. 2010). In the same way, multiple flavonoids have been also identified, mostly flavonols and flavan-3-ols, like rutin and catechin from *Porphyra dentata* (Kazłowska et al. 2010), and quercetin, rutin and catechin from *Euchema cottonii* (Namvar et al. 2012). Despite being reported as exclusive of brown algae, phlorotannins have been also recently associated with red algae (Aziz et al. 2020). These polymers of phloroglucinol have caught the attention of many researchers due to their function as bioactive compounds restricted to marine sources. On the contrary, little is known about bromophenols, which contribute to seaweed flavor but have been also reported as secondary metabolites, thus requiring further studies aiming at their characterization as bioactive compounds (Cotas et al. 2020). Besides phenolics, another compounds proceeding from the polyketide biosynthetic pathway are furanones, which have been largely determined in different red algae combining their involvement in settlement and their effectiveness as bioactive compounds (Dworjanyn et al. 2006).

In the case of terpenoids, several compounds with different isoprene polymerization degree have been found in red seaweeds, ranging from sesquiterpenoids to tetraterpenoids. Thus, in the case of red algae, most terpenoids are biosynthesized in response to the attack of herbivores and pathogenic microorganisms (Philippus et al. 2018). Nevertheless, on top of these compounds, carotenoids are considered one of the major terpenoids found in red algae, also contributing to their special pigmentation, being mainly represented by α- and β-carotene, lutein, and zeaxanthin. Among them, β-carotene gained much interest in the field of food industry because of its behavior as a natural colorant and antioxidant, being suggested as a promising candidate for its addition to food matrices (Holdt and Kraan 2011).

With respect to alkaloids, limited information is available in seaweed sources from the literature. However, previous evidence has pointed at these nitrogen-containing compounds as excellent anti-inflammatory compounds of marine origin, which prompted the research on their isolation and characterization (Souza et al. 2020). In this sense, red algae from *Gracilaria* genus have been identified as excellent sources of these marine alkaloids, together with those from *Laurencia* genus in a lesser extent, whose anti-inflammatory and antimicrobial mechanisms of action have been widely characterized, being azocinyl morpholinone the major compound (de Almeida et al. 2011).

## Biological properties

### Antioxidant activity

Several reports have indicated that extracts derived from different red algae species promote a potent antioxidant activity throughout different mechanisms, including free-radical and reactive oxygen species (ROS) scavenging activity, inhibition of lipid oxidation, and metal chelation. It is important to highlight that this activity has been proved by different assays, showing a strong dependence on the species and the experimental procedure employed for the performance of plant extracts (Rodrigues et al. 2015c). Moreover, among the different compounds isolated from red seaweeds, phenolic compounds, especially phenolic acids and flavonoids, and sulphated polysaccharides, mostly carrageenans, have been identified as the major responsible of the antioxidant activity associated with these species. Table 2 shows an overview of the antioxidant activity determined in different red algal extracts. Thus, the phenolic compounds-enriched extracts from different Rodophyta species were assessed in terms of free-radical scavenging activity (RSA), as determined by 2,2-diphenyl-pycril-hydrazyl (DPPH), and hydrogen peroxide-mediated 2,2-azinobis-(3-ethylbenzothiazoline-6-sulfonate) (ABTS) oxidation of the extracts from *Porphyra tenera* (Onofrejová et al. 2010), *Gracilaria verrucosa* (de Almeida et al. 2011), *Gracilaria arcuata* (Agatonovic-Kustrin and Morton 2017), and *Palmaria palmata* (Wang et al. 2010; Hardouin et al. 2014). In the same way, polysaccharides from red algae have been revealed as potent antioxidant compounds, according to the results found for the extracts from *Mastocarpus stellatus* (Gómez-Ordóñez et al. 2014) and *Pterocladia capillacea* (Fleita et al. 2015), by means of DPPH scavenging. On the other hand, the antioxidant properties of red algal extracts in terms of metal chelation and reducing power were assessed via the determination of ferric reduction antioxidant power assay (FRAP) and the ferrous chelating ability determination, being reported in extracts derived from *Gracilaria birdiae* (Fidelis et al. 2014), *M. stellatus* (Gómez-Ordóñez et al. 2014), and *P. palmata* (Yuan et al. 2005; Wang et al. 2010; Hardouin et al. 2014). Once again, both phenolic compounds and carrageenans have been suggested as responsible for the development of such bioactivity (Table 2). Furthermore, both compounds were reported to scavenge ROS, shown by the Oxygen Radical Absorbance Capacity (ORAC) determination, and inhibit lipid peroxidation through the thiobarbituric acid reactive substances assay (TBARS), as recorded in extracts from different species, such as *G. birdiae* (Fidelis et al. 2014), *Porphyra yezoensis* (Zhou et al. 2012), *Gigartina* spp. (Rocha De Souza et al. 2007), *P. palmata* (Yuan et al. 2005; Wang et al. 2010; Hardouin et al. 2014) and *Soliera chordalis* (Suwal et al. 2019).

**Table 2:** Antioxidant and anti-inflammatory properties of red algae

Species	Extraction (solvent)	Compound	Activity	References
Antioxidant				
*Porphyra tenera*	PLE (50% MeOH)	PA	TEAC = 20–25 µmol/g	Onofrejová et al. (2010)
*Gracilaria birdiae*	UAE (0.1 M NaOH)	SP	Total antioxidant capacity = 41.6–75.9 mg/g AAE	Fidelis et al. (2014)
*Porphyra yezoensis*	UAE (W)	PF	Scavenging activity on hydroxyl (0.065 mg/mL) and superoxide radical (0.182 mg/mL)	Zhou et al. (2012)
*Mastocarpus stellatus*	SLE (W)	Carrageenans	FRAP (44.9 μmol TE/g)	Gómez-Ordóñez et al. (2014)
*Gigartina* spp*.*	EAE (alkaline protease)	Fucoidan	Inhibition of superoxide radical (IC_50_ = 0.058 mg/mL). Peroxidation (IC_50_ = 1.250 mg/mL)	Rocha De Souza et al. (2007)
*Gracilaria arcuata*	SLE (EtOH)	PC, sterols	DPPH (27.3 GAE mg/100 g)	Agatonovic-Kustrin and Morton (2017)
*Palmaria palmata*	EAE (proteases & cellulases, W)	TPC	DPPH (EC_50_ = 0.6–1.9 mg/mL) ORAC (35.8 mmol TE/g extract)	Wang et al. (2010)
*Pterocladia capillacea*	EAE (glucanase & galactosidase)	PF	DPPH (Top value = 91.5% at 1000 mg/mL)	Fleita et al. (2015)
*Palmaria palmata*	HHPE + EAE (polysaccharidases)	TPC	ORAC (4–12 μg TE/g)	Suwal et al. (2019)
*Solieria chordalis*		PF, Proteins	ORAC (15–20 μg TE/g)	
Anti-inflammatory				
*Gracilaria caudata*	HAE (W)	SP	MPO activity, CKs levels reduction	Chaves et al. (2013)
*Solieria filiformis*	SLE (0.1 M NaCOOH buffer, papain digestion)	SP	Inhibition of nociceptive effects	De Araújo et al. (2011)
*Chondrus verrucosus*	HAE (0.17 M HCl)	SP	Inhibition of RBL-2H3 cell line	He et al. (2019)
*Gelidium pacificum*	HAE, (W, 95% EtOH)	SP	Inhibition o NO production from LPS-induced THP-1 cell line	Cui et al. (2019)
*Gracilaria salicornia*	SLE (EtOAc:MeOH, 1:1)	Chromenyl compounds	Inhibition of anti-inflammatory enzymes: COX-2, 5-LOX	Antony and Chakraborty (2019)
*Gracilaria birdiae*	SLE (0.1 M NaCOOH buffer, papain digestion)	SP	Inhibition of HO-1 pathway	De Sousa Oliveira Vanderlei et al. (2011)
*Gracilaria cornea*	SLE (0.1 M NaCOOH buffer, papain digestion)	SP	Inhibition of nociceptive effects, neutrophil migration, and oedema	Coura et al. (2012)
*Gracilaria opuntia*	HAE (W)	SP	Inhibition of anti-inflammatory enzymes: COX-1, 5-LOX	Makkar and Chakraborty (2017)
*Kappaphycus alvarezii*	SLE (MeOH:EtOAc, 1:1)	Terpenoids	Inhibition of inflammatory enzymes: COX, LOX	Chatter et al. (2011), Makkar and Chakraborty (2018)
*Laurencia glandulifera*	n.d	Neorogioltriol (diterpenoid)	Inhibition of edema in vivo, activity against LPS-induced macrophages, inhibition of NF-kB activation, TNF-α and NO levels and COX-2	Chatter et al. (2011), Makkar and Chakraborty (2018)
*Palmaria palmata*	SLE (MeOH/CHCl_3_)	Phospholipids	Inhibition of NO production by LPS-induced macrophages	Banskota et al. (2014)
*Laurencia snackeyi*	SLE (MeOH)	Halogenated monoterpenes	CKs, TNF- α, Il-1β, and IL-6 levels reduction	Wijesinghe et al. (2014)
*Porphyra columbina*	SLE (W)	PB	Upregulation of CKs: IL-10	Cian et al. (2012)

PLE, Pressurized liquid extraction; UAE, Ultrasound assisted extraction; SLE, Solid–liquid extraction; EAE, Enzyme assisted extraction, High hydrostatic pressure extraction; HAE, Heat assisted extraction; W, Water; TPC, Total phenolic compounds; PA, Phenolic acids; PC, Phenolic compounds; PB, Phycobiliproteins; SP, Sulfated polysaccharides; PF, Polysaccharide fraction; AAE, Ascorbic acid equivalent; TE, Trolox equivalents; GAE, Gallic acid equivalent; DPPH, 2-diphenyl-1-picrylhydrazyl; ORAC, Oxygen Radical Absorbance Capacity; TEAC, Trolox equivalent antioxidant capacity; MPO, Myeloperoxidase; NO, Nitric oxide; HO-1, Hemoxygenase-1; LPS, Lipopolysaccharide; COX-2, Cyclooxygenase-2; 5-LOX, 5-lipoxygenase; CKs, Cytokines; TNF-α, Tumor necrosis factor; NF-kB, nuclear factor kappa-light-chain-enhancer of activated B cells; IL, Interleukin

### Anti-inflammatory activity

Inflammation constitutes a multifactorial physiological process, developed by the immune system, closely related to oxidative stress, and contributing to cancer onset. Consequently, greater efforts should be directed to alleviate inflammation-related phenomena. In this sense, red algae extracts have been reported to promote a multifaceted anti-inflammatory activity, as presented in Table 2, by the regulation of several phenomena, including the alleviation of inflammation-associated nociceptive effects, the inhibition of pro-inflammatory enzymes and cytokines (CKs), the inhibition of leukocyte migration, the regulation of cell signaling pathways involved in the onset of inflammation, and the promotion of anti-inflammatory CKs. Once again, sulphated polysaccharides, especially carrageenans, and proteins, such as lectins and phycobiliproteins, were assigned as the major responsible for the anti-inflammatory effects attributed to red algae (Table 2). Indeed, such effects have been demonstrated in both in vitro models, as it is the case of lipopolysaccharide (LPS)-induced RAW 264.7 macrophages, and in vivo*,* mostly murine models.

Thus, carrageenan-enriched extracts from different Rhodophyta species have been assessed in terms of anti-inflammatory effects, as it was observed by *Gracilaria caudata* (Chaves et al. 2013), *Solieria filiformis* (De Araújo et al. 2011), *Chondrus verrucosus* (He et al. 2019), *Gelidium pacificum* (Cui et al. 2019), different species from *Gracilaria* genus (*Gracilaria salicornia* (Antony and Chakraborty 2019), *G. birdiae* (De Sousa Oliveira Vanderlei et al. 2011), *G. cornea* (Coura et al. 2012), and *Gracilaria opuntia* (Makkar and Chakraborty 2017)). Among the different effects attributed to these extracts, the most relevant pro-inflammatory enzymes inhibited were cyclooxygenases-1 and -2 (COX) and lipoxygenase (5-LOX) and myeloperoxidase. In addition, lipid-enriched extracts from *Kappaphycus alvarezii* [92], *Laurencia grandulifera* [93], *P. palmata* (Banskota et al. 2014), and *Laurencia snackeyi* (Wijesinghe et al. 2014) enabled the reduction of the expression of pro-inflammatory CKs *i.e.*: tumor necrosis factor alpha (TNF-α), and interleukins (ILs) 1β and 6, the inhibition of nitric oxide (NO) production. Additionally, the phycobiliprotein-enriched extracts of *Porphyra columbina* were recorded in the basis of the up-regulation of anti-inflammatory CKs, such as IL-10 (Cian et al. 2012).

In general, the combined determination of antioxidant, anticancer, and anti-inflammatory activities from red seaweed extracts could face the further determination of these species as promising natural sources of cancer chemopreventive agents (García-Pérez et al. 2019).

### Metabolic activity

Nowadays, diabetes and cardiovascular diseases are one of the most important global health problems, since they are the main responsible for premature deaths between 30 and 70 years, together with cancer (World Health Organization 2019). In this context, numerous studies have pointed at red macroalgae as natural sources of compounds devoted to the prevention and treatment of metabolic and chronic diseases, as it is the case of diabetes and obesity. Several authors have highlighted the existence of multiple compounds isolated from red macroalgae with the ability of regulating the hyperglycemia caused by diabetes, as reported by both in vivo and in vitro models*,* indicating that red algae are interesting candidates for the development of novel drugs for the treatment of this metabolic disorder (Ezzat et al. 2018). Thus, different mechanisms of actions have been proposed for the anti-diabetic effects of red algae extracts, as shown in Table 3. In summary, three major mechanisms have been described for the anti-diabetic activity of red algae extracts, including the inhibition of insulin cell-signaling repressors, such as protein tyrosine phosphatase 1B (PTP1B) (Wang et al. 2015b), reduction of circulating glucose levels, and the inhibition of saccharidases, involved in the synthesis of free monosaccharides, as it is the case of α-amylase, α-glucosidase, aldose reductase or dipeptidyl peptidase-4 (DPP4) (Table 3). Among the different compounds from red algae responsible for such bioactivity, bromophenols play a fundamental role, on top of other molecules, like sulphated polysaccharides and proteins.

**Table 3:** Antidiabetic and lipid metabolism related activities of red algae

Species	Extraction (solvent)	Compound	Activity	References
Antidiabetic				
*Rhodomela confervoides*	Synthetic derivation	Bromophenols	Inhibition of PTP1B in vitro and in vivo	Shi et al. (2012)
*Odonthalia corymbifera*	Isolation of bis(2,3-dibromo-4,5-dihydroxybenzyl) ether	Bromophenols	Inhibition of PTP1B in vitro and in vivo	Xu et al. (2016)
*Symphyocladia latiuscula*	SLE (95% EtOH)	Bromophenols	Inhibition of PTP1B in vitro	Liu et al. (2011)
*Kappaphycus alvarezii/ Gracilaria opuntia*	HAE (W)	SP	Inhibition of α-amylase, α -glucosidase and DPP-4	Makkar and Chakraborty (2017)
*Symphyocladia latiuscula*	SLE (95% EtOH)	Bromophenols	Inhibition of aldose reductase	Wang et al. (2005)
*Polyopes lancifolia*	SLE (80% MeOH)	Bromophenols	Inhibition of α -glucosidase, sucrase and maltase	Kim et al. (2010)
*Grateloupia elliptica*	SLE (75% MeOH)	Bromophenols	Inhibition of α -glucosidase	Kim et al. (2008)
*Palmaria palmata*	SLE (W, alkaline hydrolysis 0.12 M NaOH)	Protein hydrolysate	Inhibition of DPP-4	Harnedy and FitzGerald (2013)
Lipid metabolism				
*Gracilaria changii*	Powdered, directly administered to animals	Whole algae	Reduction of plasma levels of TC, LDL-C, TAG and atherogenic index	Chan et al. (2014)
*Kappaphycus alvarezii*	Powdered, directly administered to animals	Whole algae	Reduction of plasma levels of TC, LDL-C, TAG, lipid peroxidation, increase of HDL levels	Matanjun et al. (2010)
*Gigartina pistillata*	Powdered, directly administered to animals	Whole algae	Reduction of plasma levels of TC, LDL-C, TAG, and hepatic TAG levels	Villanueva et al. (2014)
*Porphyra tenera*	Powdered, directly administered to animals	Whole algae	Reduction of plasma levels of TC	Bocanegra et al. (2008)
*Porphyra umbilicalis*	Powdered, directly administered to animals	Whole algae	Reduction of plasma level of TC, alleviation of obesity-related oxidative stress	Moreira et al. (2010)
*Melanothamnus afaqhusainii*	SLE (EtOH)	SP	Reduction of plasma levels of TC, LDL-C, TAG, increase of HDL-C levels	Ruqqia et al. (2014)
*Porphyra yezoensisc*	HAE (W)	SP	Increase of fecal excretion of cholesterol	Tsuge et al. (2004)
*Porphyra* sp.	Porphyran isolation	SP	Reduction of ApoB100 levels in vitro	Inoue et al. (2009)
*Prophyra haitanensis*	Oxidative degradation	SP	Reduction of TC, TC and LDL-C, increase of HDL-C	Wang et al. (2017)

SLE, Solid–liquid Extraction; HAE, Heat Assisted Extraction; W, Water; SP, Sulfated polysaccharides; PTP1B, protein-tyrosine phosphatase 1B; DPP4, Dipeptidyl peptidase-4; TC, total cholesterol; LDL-C, low density lipoprotein cholesterol; TAG, triglycerides; HDL-C, high density lipoprotein cholesterol

Thus, bromophenols have been reported as multifaceted antidiabetic agents, developing different mechanisms which include the inhibition of PTP1B by both in vivo and in vitro models, as found for *Rhodomela confervoides* (Shi et al. 2012), *Odonthalia corymbifera* (Xu et al. 2016), and *Symphyocladia latiuscula* (Liu et al. 2011), and the enzymatic inhibition of α-glucosidase by *Polyopes lancifolia* and *Grateloupia elliptica* extracts (Kim et al. 2008, 2010), and aldose reductase by *S. latiuscula* extracts (Wang et al. 2005). Besides bromophenols, the inhibition of enzymes related with type-2 diabetes has been reported to sulphated polysaccharides from *K. alvarezii* and *G. opuntia* extracts, exhibiting a potent inhibition of α-amylase, α-glucosidase, and DPP-4 (Makkar and Chakraborty 2017), and the protein hydrolysate from *P. palmata*, acting as inhibitor of DPP-4 (Harnedy and FitzGerald 2013).

Concerning anti-hyperlipidemic effects of red seaweed extracts, strong evidence has been reported on rodent in vivo models, as well as in vitro systems. Therefore, the dietary administration of several species, such as *Gracilaria changii* (Chan et al. 2014), *K. alvarezii* (Matanjun et al. 2010), *Gigartina pistillata* (Villanueva et al. 2014), *P. tenera* (Bocanegra et al. 2008), and *P. umbilicalis* (Moreira et al. 2010) on hypercholesterolemic rodent models have promoted the reduction in the plasmatic levels of total cholesterol (TC), low-density lipoprotein cholesterol (LDL-C), and triacylglycerols (TAG), as well as the increase in high-density lipoprotein cholesterol (HDL-C). Moreover, red algal extracts were shown to decrease the hepatic accumulation of cholesterol, reduce the atherogenic index, inhibit lipid peroxidation, and alleviate the obesity-related oxidative stress in the same in vivo models (Chan et al. 2015; Patil et al. 2018). With respect to individual compounds, sulphated polysaccharides, especially porphyran, were revealed as the major responsible of the above-mentioned mechanisms, together with the increase on fecal excretion of cholesterol and the reduction of the apolipoprotein B 100 level in vitro, as found for *Melanothamnus afaqhusainii* (Ruqqia et al. 2014) and several *Porphyra* species (Inoue et al. 2009), such as *P. yezoensis* (Tsuge et al. 2004) and *P. haitanensis* (Wang et al. 2017). On these bases, carrageenans were also reported for their hypocholesterolemic properties (Panlasigui et al. 2003). Keeping all this in mind, the consumption of red seaweeds can be regarded as a beneficial approach to alleviate the physiological complications attributed to chronic metabolic diseases, such as type-2 diabetes and hypercholesterolemia, with positive implications on the development of currently critical diseases, as it is the case of obesity and cardiovascular diseases.

### Antitumor activity

Seaweed secondary metabolites have been reported to show antitumor activity, thus showing the potential of a novel source of natural pharmaceuticals (Ahmed et al. 2011). This is the case of halogenated metabolites and sulphated polysaccharides, mostly. Such efficiency, as antitumor agents from red seaweeds, has been assessed towards a plethora of cancer cell lines from human neoplastic diseases. Table 4 contains a summary of the different studies committed to the description of anticancer activity of red algae extracts.

**Table 4:** In vitro and in vivo antitumor activity of red algae

Species	Compound	Cell line/Animal	Solvent	Activity	References
In vitro experiments					
*Callophycus serratus*	Bromophycolide H	DU4475	A:W	IC_50_ = 3.88 μM	Kubanek et al. (2006)
*Champia feldmannii*	SP	HL-60, MDA-MB-435, SF-295, HCT-8	CPC	IC_50_ = > 25 µg/mL	Lins et al. (2009)
*Chondria atropurpurea*	Chondriamide-A	KB and LOVO cells	E:C	n.d	Palermo et al. (1992), Smit (2004)
*Eucheuma cottonii*	Polyphenols	MCF-7 // MB-MDA-231	M	IC_50_ = 20 // 42 µg/mL	Namvar et al. (2012)
*Euchema serra*	Agglutinin (lectin)	Colo201, HeLa // OST, LM8	E	IC_50_ =–// 50 µg/mL	Sugahara et al. (2001), Hayashi et al. (2012)
*Galaxoura cylindriea*	Sulfolipids	Hep G2 // MCF-7	C:M	IC_50_ = 2.75 // 0.40 µg/mL	El Baz et al. 2013)
*Gelidium amansii*	SP (agar)	Hepa-1, HL-60, NIH-3T3	M	n.d	Chen et al. (2004)
*Gloiopeltis furcate*	SP	MKN45 and DLD-1	W	< 25% and 19.07% inhibition	Shao et al. (2013)
*Gracilaria caudata*	SP	HeLa	Maxataze (protease)	30–40% inhibition	Costa et al. (2010)
*Grateloupia elliptica*	Pheophorbide A	U87MG // SK-OV-3 // B16-BL6 // SiHa // HeLa	M	IC_50_ = 2.8 // 7.0 // 18.3 // 13.2 // 9.5 µg/mL	Cho et al. (2014)
*Hypnea muscifformis*	Kappa-carrageenan	MCF-7, SH-SY5Y	Papain, SA, CPC	50–75% proliferation inhibition	Souza et al. (2018)
*Jania rubens*	SP	CoCa2 // MCF7	W:E	IC_50_ = 20 // 0.3125 mg/mL	Gheda et al. (2018)
*Laurencia microcladia*	Elatol	L929 > DU145 > MCF7 > A549 > B16F10	E	IC_50_ = Max. 1.1 μM (L929). Min. 10.1 μM (B16F10)	Campos et al. (2012)
*Laurencia obtusa*	Brominated diterpenes	MCF-7 // PC3 // HeLa // A431 // K562	C:M	IC_50_ = 149.5 // 138 // 78.4 // 86.2 // 108.3 µM	Iliopoulou et al. (2003)
*Laurencia popillose*	Sulfolipids	Hep G2 // MCF-7	C:M	2.21 // 0.67 µg/mL	El Baz et al. (2013)
*Polysiphonia lanosa*	Major Bromophenols	DLD-1	M	IC_50_ = 39.7 ± 1.5 µg/mL	Shoeib et al. (2004)
*Solieria filiformis*	Mixture of lectins isoforms	MCF-7	PBS	IC_50_ = 125 µg/mL	Chaves et al. (2018)
*Sphaerococcus coronopifolius*	Sphaerococcenol A (Bromoditerpene)	HepG-2	DCM	IC_50_ = 42.87 µg/mL	Rodrigues et al. (2015a)
	Bromoditerpenes	U373, A549, SKMEL-28, OE21, PC3, LOVO	DCM:M	IC_50_ = 3–76 μM	Smyrniotopoulos et al. (2010)
In vivo experiments					
*Champia feldmannii*	SP + 5-Fu	Swiss mice (25–30 g) subc. S180	CPC	Dose = 10 + 10 mg/kg. TVR = (48–68%)	Lins et al. (2009)
*Chondrus ocellatus*	Low MW λ-Carrageenan + 5-Fu	65 ICR mice (20 g) transplanted subc. S180	W:E	Dose = 100 + 25 mg/kg TVR = (63%)	Zhou et al. (2005)
	Low MW λ-Carrageenan + 5-Fu	65 ICR mice (20 g) transplanted with H-22	W:E	Dose = 100 + 25 mg/kg TVR = (52%)	Zhou et al. (2006)
*Eucheuma cottonii*	Polyphenols	Rats (200–250 g) subc. LA7	M	Dose = 150–300 mg/kg (37%)	Namvar et al. (2012)
*Laurencia microcladia*	Elatol	C57BL6 mice (18–25 g)	E	Dose = 10 mg/kg TVR = (71%)	(Campos et al. 2012)

B16F10 (Murine melanoma number CR-010), A549 (human lung carcinoma), DU145 (human prostate carcinoma), L929 (murine fibroblast), Hepa-1 (murine hepatoma), HL-60 (human leukemia), NIH-3T3 (murine embryo fibroblast cells), MB-MDA-231 (human breast cancer cell), LA7 (aka, CRL 2283: rat mammary gland tumor cell line), MKN45 (gastric cancer cells), MDA-MB-435 (Melanoma), SF-295 (glioblastoma), HCT-8 (Human colon), DLD-1 (human colon adenocarcinoma), Colo201 (human colon adenocarcinoma), HeLa (human cervix adenocarcinoma), MCF-7 (human breast adenocarcinoma), HB4C5 cells (human hybridoma cell line), OST (Human osteosarcoma Takase cells), LM8 cells (Murine osteosarcoma cell line), DU4475 (breast tumor cell line), PC3 (prostate adenocarcinoma), A431 (derived from epidermoid carcinoma), K562 (chronic myelogenous leukemia cell line), SkMel28 (human malignant melanoma), CHO (Chinese hamster ovary cells), SH-SY5Y (human neuroblastoma), Caco-2 (colon cancer cell line), U87MG (Human glioblastoma cells), B16-BL6 (mouse melanoma cells), SiHa (human cervical cancer cells), SKOV-3 (human ovarian cancer cells), OE21 (oesophageal squamous cell carcinoma), TVR (Tumor volume reduction), A (Acetone), W (Water), CPC (Cetylpiridinium chloride), C (Chloroform), E (Ethanol), SA (Sodium acetate), M (Methanol), PBS (phosphate buffered saline), DCM (Dichloromethane), ip. (intraperitoneal), subc. (subcutaneously), S180 (Sarcoma-180 tumor cells), H-22 (mouse hepatocellular carcinoma), LA7 (rat breast cancer stem cells)

Different authors have pointed at brominated compounds from *Callophycus serratus* (Kubanek et al. 2006) *Laurencia obtusa* (Iliopoulou et al. 2003), *Plocamium cartilagineum* (De Inés et al. 2004), *Polysiphonia lanosa* (Shoeib et al. 2004), *Portieria hornemanii* (Fuller et al. 1992), and *Sphaerococcus coronopifolius* (Smyrniotopoulos et al. 2010; Rodrigues et al. 2015a). As a result, a panel of diverse cancer cell lines has been proved to be affected by halogenated compounds-enriched extracts, including leukemia, lung, breast, colon, and cervix cancer cell lines. Accordingly, sulphated polysaccharides, such as carrageenans, have been also reported as efficient anticancer agents, being conducted in different red seaweed species, *i.e.*: *Champia feldmannii* (Lins et al. 2009), *Gelidium amansii* (Chen et al. 2004; Shao et al. 2013), *Gracilaria caudata* (Costa et al. 2010), *Hypnea mascifformis* (Souza et al. 2018), and *Jania rubens* (Gheda et al. 2018). In a lesser extent, polyphenols from *E. cottonii* (Namvar et al. 2012) extracts, and lectins from *Euchema serra* (Sugahara et al. 2001; Hayashi et al. 2012) and *S. filiformis* (Chaves et al. 2018) have been reported in the basis of their antitumor activity.

The anticancer activity reported on red algae extracts have been conducted under in vitro conditions and little information on in vivo models is currently available. However, some studies have proven the activity of certain compounds in tumor volume reduction (TVR) (Campos et al. 2012; Namvar et al. 2012) and in combination with 5-Fluoroacil, a drug used in chemotherapy (Zhou et al. 2005, 2006; Lins et al. 2009). Also, similar percentages of TVR were reported when using elatol extracted from *Laurencia microcladia* (71%) compared to positive control cisplatin (81%) (Campos et al. 2012) (Table 4). Further studies are required, in this sense, to ensure their effectiveness in humans, and molecular insights are equally desired to elucidate the specific mechanism of action of anticancer compounds, to facilitate their consideration as official food and pharmaceutical ingredients.

### Antimicrobial activity

Antimicrobial activity has been attributed to different compounds derived from red seaweeds, being regarded as effective antibacterial, antifungal, and antiviral compounds, especially glycolipids, lectins, terpenoids and furanones, as well as different halogenated metabolites. Table 5 shows an overview of the effectiveness of red algae extracts as sources of antimicrobial compounds. Concerning antibacterial activity, several red algae species have been shown to promote a relevant effectiveness towards both Gram + and Gram—bacteria, as it is the case of *C. crispus, Gelidium latifolium, P. palmata, Ceramium rubrum, Cryptopleura ramosa, Laurencia pinnatifida* and *Polysiphonia lanosa* (Hellio et al. 2001)*.*

**Table 5:** Antimicrobial activity of red algae

Species	Compound	Solvent	Microorganism	References
*Alsidium corallinum*	ns	M	*E. coli, K. pneumoniae, S. aureus*	Rhimou et al. (2010)
*Amphiroa rigida*	ns	M	*S. aureus*	Val et al. (2001)
*Asparagopsis taxiformis*	ns	M	*B. subtilis, E. faecium, M. smegmatis, P. aeruginosa, S. marcescens, S. aureus, S. cerevisiae, C. albicans, A. fumigatus*	Val et al. (2001)
*Callophycus serratus*	Bromophycolide	W, M, DCM	*VREF, MRSA*	Lane et al. (2009)
*Ceramium rubrum*	ns	M	*E. coli, E. faecalis, S. aureus*	Rhimou et al. (2010)
*Ceramium virgatum*	Fatty acids	EE:H	*B. cereus, E. coli, L. monocytogenes, S. enteriditis*	Horincar et al. (2014)
*Chondria armata*	Glycolipis	M	*Klebsiella* sp., *C. albicans, A. fumigatus, C. neoformans*	Al-Fadhli et al. (2006)
*Chondrocanthus acicularis*	ns	M	*E. coli, E. faecalis, K. pneumoniae, S. aereus*	Rhimou et al. (2010)
*Chondrus crispus*	ns	E, EA	*B. cereus, C. marina, E. coli, E. faecalis, H. marina, L. brevis, L. innocua, M. hydrocarbonoclasticus, P. aeruginosa, P. elyakovii, P. irgensii, S. enteriditis, S. putrefaciens, S. aureus, V. aestuarianus, Candida* sp.	Chambers et al. (2011; Mendes et al. (2013; Salta et al. (2013)
*Corallina elongata*	Lipids	A, M or E	*B. subtilis, E. coli, K. pneumoniae, S. typhi, S. aureus, C. albicans*	Val et al. (2001), Osman et al. (2010)
*Corallina mediterranea*	1,2-BDC	M, E	*V. fluvialis*	Mohy El-Din and El-Ahwany (2016)
*Delisea pulchra*	Furarones	95% E	*E. coli, P. aeruginosa*	Manefield et al. (2001), Ren et al. (2004)
	Furanones, catechins	n.d	*C. jejuni*	Castillo et al. (2015)
*Euchema serra*	Lectins	W:E	*V. pelagius, V. vulnificus*	Liao et al. (2003)
*Falkenbergia-phase of A. taxiformis*	Volatile compounds	M	*B. subtilis, K. pneumoniae, P. aeruginosa, S. aureus, S. epidermidis, V. alcaligenes, V. alginolyticus*	Manilal et al. (2010)
*Galaxaura marginata*	Lectins	W/E	*V. neresis, V. pelagius, V. vulnificus*	Liao et al. (2003)
*Galaxaura rugosa*	ns	M	*B. subtilis*	Val et al. (2001)
*G. rugosa*	ns	M	*B. subtilis*	Val et al. (2001)
*Gelidium arbuscula*	ns	M	*B. subtilis*	Val et al. (2001)
*Gelidium attenatum*	ns	M	*E. coli, E. faecalis, K. pneumoniae, S. aureus*	Rhimou et al. (2010)
*Gelidium micropterum*	ns	M	*V. alcaligenes, V. parahaemolyticus*	Manilal et al. (2010)
*Gelidium pulchellum*	ns	M	*E. coli, E. faecalis, S. aureus*	Rhimou et al. (2010)
*Gelidium pusillum*	ns	M	*E. coli, E. faecalis, K. pneumoniae, S. aureus, V. alcaligenes, V. alginolyticus, V. harveyi, V. parahaemolyticus, V. vulnificus*	Manilal et al. (2010), Rhimou et al. (2010)
*Gelidium spinulosum*	ns	M	*E. coli, E. faecalis, S. aureus*	Rhimou et al. (2010)
*Gracilaria corticata*	ns	M or DMSO	*B. subtilis, E. coli, P. fluoresens, S.aureus*	Arulkumar et al. (2018)
*Gracilaria dura*	Lipids	C:M (2:1)	*V. alginolyticus, V. ordalii*	Cavallo et al. (2013)
*Gracilaria edulis*	ns	EA, M or DMSO	*A. hydrophyla, B. subtilis, E. coli, P. fluoresens, S.aureus, V. fluvialis*	Arulkumar et al. (2018), Kasanah et al. (2019)
*Gracilaria fisheri*	Proteins	25 mM Tris–HCl	*V. harveyi, V. parahaemolyticus*	Boonsri et al. (2017), Karnjana et al. (2019)
*Gracilaria gracilis*	Lipids	C:M (2:1)	*V. salmonicida*	Cavallo et al. (2013)
*Gracilaria ornata*	SP	W	*E. coli*	dos Santos Amorim et al. (2012)
*Gracilaria vermiculophylla*	ns	E or EA	*B. subtilis, E. coli, E. faecalis, L. brevis, L. innocua, P. aeruginosa, S. enteriditis, S. aureus, Candida* sp.	Mendes et al. (2013)
*Gracilariopsis longissima*	Lipids	C:M (2:1)	*V. alginolyticus, V. cholerae non O-1, V. fluvialis, V. ordalii, V. salmonicida, V. vulnificus*	Stabili et al. (2012), Cavallo et al. (2013)
*Grateloupia livida*	ns	E or M	*B. subtilis, E. coli, P. aeruginosa, S. aureus, V. alcaligenes, V. alginolyticus, V. harveyi, V. parahaemolyticus, V. vulnificus*	Manilal et al. (2010), Jiang et al. (2013), Kavita et al. (2014)
*Haliptilon virgatum*	ns	M	*S. aureus*	Val et al. (2001)
*Halopitys incurvus*	ns	M	*B. subtilis, E. coli, E. faecalis, K. pneumoniae, S. aureus*	Val et al. (2001), Rhimou et al. (2010)
*Hypnea musciformis*	Agglutinins, kappa carrageenan	M or PBS:W	*E. coli, E. faecalis, K. pneumoniae, S. aureus, C. albicans, T. rubrum, C. lindemuthianum*	Melo et al. (1997), Rhimou et al. (2010), Souza et al. (2018)
*Hypnea pannosa*	ns	M	*B. subtilis, E. coli, P. aeruginosa, S. aureus*	Kavita et al. (2014)
*Hypnea valentiae*	ns	E	*B. subtilis, E. coli, P. aeruginosa, S. aureus, S. pyogenes*	Rhimou et al. (2010), Kavita et al. (2014)
*Jania rubens*	Lipids/1,2-BDC	A, M, E, DCM or C	*B. cereus, B. subtilis, E. cloacae, E. coli, E. faecalis, K. pneumoniae, P. aeruginosa, S. typhi, S. typhimurium, S. aureus, S. epidermis, V. fluvialis, C. albicans*	Horzum et al. (2006), Osman et al. (2010), Mohy El-Din and El-Ahwany (2016)
*Laurencia majuscula*	Halogenated acetogenins	M	*E. coli, K. pneumoniae, Pseudomonas* sp.*, Salmonella* sp*., S. aureus, S. epidermis*	Vairappan et al. (2001)
*Laurencia papillosa*	Sulfolipids	M:C (2:1)	*B. subtilis, E. coli, K. pneumoniae, P. aeruginosa, S. flexneri, S. aureus.* Antiviral against HSV-1	El Baz et al. (2013), Kavita et al. (2014)
*Laurencia* spp.	Halogenated acetogenins	M	*Clostridium* spp. *and P. mirabilis*	Manilal et al. (2010)
*Liagora farinosa*	ns	M	*B. subtilis, S.aureus*	Val et al. (2001)
*Osmundea hybrida*	ns	M	*B. subtilis, M. smegmatis, S. aureus, S. cerevisiae*	Val et al. (2001)
*Palmaria palmata*	ns	M	*E. faecalis, L. monocytogenes, P. aeruginosa, S. abony*	Rudtanatip et al. (2018)
*Plocamium cartilagineum*	ns	M	*E. coli, E. faecalis, S. aureus*	Rhimou et al. (2010)
*Polysphonia tuticorinensis*	ns	M	*B. subtilis, E. coli, P. aeruginosa, S. aureus*	Kavita et al. (2014)
*Porphyra dioica*	ns	E or EA	*B. cereus, E. coli, E. faecalis, L. brevis, S. aureus, Candida* sp.	Mendes et al. (2013)
*Porphyra umbilicalis*	Fatty acids	H, W or 80% W:M	*P. digitatum, B. cinerea, M. laxa*	De Corato et al. (2017)
*Porphyra yezoensis*	Peptide	Pepsin digestion	*S. aureus*	Jiao et al. (2019)
*Portieria horemanii*	ns	M	*V. alginolyticus, V. harveyi, V. vulnificus*	Manilal et al. (2010)
*Pterocladia capillacea*	Lipids/Lectins/1,2-BDC	A, M or E	*B. cereus, B. subtilis, E. coli, K. pneumoniae, P. fluoresens, S. typhi, S. aureus, S. pyogenes, V. fluvialis, V. pelagius, V. vulnificus, C. albicans, F. oxysporium*	Liao et al. (2003), Osman et al. (2010), Abou Zeid et al. (2014), Mohy El-Din and El-Ahwany (2016)
*Pterosiphonia complanata*	ns	M	*E. coli, E. faecalis, S. aureus*	Rhimou et al. (2010)
*Solieria filiformis*	Lectins	20 mM Tris–HCl	*E. aerogenes, K. pneumoniae, P. aeruginosa, Proteus* spp., *S. typhi, S. marcescens*	Holanda et al. (2005)
*Sphaerococcus coronopifolius*	Bromoditerpene	M or DCM	*E. coli, P. aeruginosa, S. aureus*	Smyrniotopoulos et al. (2008; Rodrigues et al. (2015a)

*Escherichia coli (E. coli), Klebsiella pneumoniae (K. pneumoniae), Staphylococcus aureus (S. aureus), Bacillus subtilis (B. subtilis), Enterococcus faecium (E. faecium), Mycobacterium smegmatis (M. smegmatis), Pseudomonas aeruginosa (P. aeruginosa), Serratia marcescens (S. marcescens), Saccharomyces cerevisiae (S. cerevisiae), Candida albicans (C. albicans), Aspergillus fumigatus (A. fumigatus), Vancomycin-resistant Enterococcus faecium (VREF), Methicillin-resistant Staphylococcus aureus (MRSA), Enterococcus faecalis (E. faecalis), Bacillus cereus (B. cereus), Listeria monocytogenes (L. monocytogenes), Salmonella enteriditis (S. enteriditis), Cryptococcus neoformans (C. neoformans), Cobetia marina (C. marina), Halomonas marina (H. marina), Lactobacillus brevis (L. brevis), Listeria innocua (L. innocua), Marinobacter hydrocarbonoclasticus (M. hydrocarbonoclasticus), Pseudoalteromonas elyakovii (P. elyakovii), Polaribacter irgensii (P. irgensii), Shewanella putrefaciens (S. putrefaciens), Vibrio aestuarianus (V. aestuarianus), Salmonella typhi (S. typhi), Vibrio fluvialis (V. fluvialis), Vibrio pelagius (V. pelagius), Vibrio vulnificus (V. vulnificus), Staphylococcus epidermidis (S. epidermidis), Vibrio alcaligenes (V. alcaligenes), Vibrio alginolyticus (V. alginolyticus), Vibrio neresis (V. neresis), Pseudomonas fluoresens (P. fluoresens), Vibrio ordalii (V. ordalii), Aeromonas hydrophyla (A. hydrophyla), Vibrio salmonicida (V. salmonicida), Vibrio cholerae non O-1 (V. cholerae non O-1), Trichophyton rubrum (T. rubrum), Collectotrichum lindemuthianum (C. lindemuthianum), Enterobacter cloacae (E. cloacae), Salmonella typhimurium (S. typhimurium), Shigella flexneri (S. flexneri), Mycobacterium smegmatis (M. smegmatis), Salmonella abony (S. abony), Penicilium digitatum (P. digitatum), Botrytis cinerea (B. cinerea), Monilinia laxa (M. laxa), Fusarium oxysporium (F. oxysporium), Enterobacter aerogenes (E. aerogenes), Serratia marcescens (S. marcescens).* 1,2-Benzenedicarboxylic (1,2-BDC), ns: not specified, A (Acetone), W (Water), C (Chloroform), E (Ethanol), M (Methanol), PBS (phosphate buffered saline), DCM (Dichloromethane), EE (Ethyl ether), H (Heptane), EA (Ethyl acetate)

For instance, halogenated acetogenins of genus *Laurencia* have shown a multifaceted antimicrobial activity against a wide range of bacteria, including those from *Clostridium* and *Salmonella* genera, as well as other pathogenic species, such as *Proteus mirabilis* and *Klebsiella pneumoniae* (Vairappan et al. 2001; Vairappan 2003). Regarding the fractions rich in polar glycolipids from the algae *Chondria armata*, these compounds exhibited not only a potent antimicrobial activity against *Klebsiella* sp., but also a relevant antifungal activity against *Candida albicans* and *Cryptococcus neoformans* (Al-Fadhli et al. 2006). Concerning other lipidic substances, extracts from *Laurencia papillosa* and *Galaxoura cylindriea* enriched with sulpholipids were effective against *Escherichia coli* and *Bacillus subtilis*, as well as antiviral activity against herpes simplex virus-1 (HSV-1) (El Baz et al. 2013). Moreover, fatty acids from *Gracilaria edulis* extracts promoted an intense effectiveness against marine pathogens, such as those of *Vibrio* sp., thus contributing to the prevention of infectious diseases in the field of aquaculture (Kasanah et al. 2019). In parallel, the combination of *Bacillus amyloliquefaciens* associated with *Laurencia papillosa* has proved to inhibit the growth of some marine vibrios and the bacteria *Aeromonas hydrophilla*, being both typical food pathogens (Chakraborty et al. 2017). Furthermore, protein extracts isolated from red algal sources have been reported as natural antibacterials too. Thus, extracts from *Gracilaria fisheri* promoted an antibacterial activity against *Vibrio parahaemolyticus*, which is considered the etiologic agent of the shrimp acute hepatopancreatic necrosis disease (Boonsri et al. 2017). Among proteins, lectins have emerged as interesting antimicrobials, as those from *Soliera filiformis*, which depict a wide range of effectiveness against different pathogens, including *Serratia marcescens*, *Salmonella typhi*, *K. pneumoniae*, *Enterobacter aerogenes*, *Proteus* sp., and *Pseudomonas aeruginosa* (Holanda et al. 2005). Not only proteins have been detected because of their role as antimicrobial, since algal peptides have also shown the same bioactivity, as reported for *Porphyra yezoensis* inhibiting the growth of *Staphyloccocus aureus* (Jiao et al. 2019). At last, furanones have been identified as another relevant family of compounds with associated antimicrobial activity from red algal sources, as demonstrated for *Delisea pulchra* extracts against *Escherichia coli* and *Campylobacter jejuni* (Manefield et al. 2001; Castillo et al. 2015).

In the case of antifungal activity (Table 5), besides the already mentioned activity of glycolipids-enriched *Chondria armata* extracts against human fungal pathogens (Al-Fadhli et al. 2006), the protein extracts from *Hypnea musciformis* showed the same effectiveness against different agricultural pathogens, such as *Trichophyton rubrum* and *Colletotrichum lindemunthianum* (Melo et al. 1997). In the same way, another crop pathogens, like *Botrytis cinerea*, *Monilinia laxa*, and *Penicillium digitatum* were inhibited by the fatty acids and polysaccharide fractions of *Porphyra umbilicalis* and related species, thus suggesting a promising effect of red seaweed extracts as preventive agents of agricultural diseases (De Corato et al. 2017).

Finally, the antiviral activity of red algal extracts has been widely reported in terms of HSV-1 growth inhibition as stated before. Generally, polysaccharides, mostly carrageenans, and lectins are the major responsible of such bioactivity (Table 5). Thus, extracts from different Rhodophyta species, such as *Gracilaria* sp., *Nothogenia fastigiata*, and *Mastocarpus stellatus* have been assessed in terms of their effectiveness against HSV-1 and HSV-2 (Baba et al. 1988; Damonte et al. 1994; De Clercq 2000; Mazumder et al. 2002; Bouhlal et al. 2010; Soares et al. 2012; Gómez-Ordóñez et al. 2014). Additionally, the same extracts reported a potent activity against cytomegalovirus, vesicular stomatitis virus, and several respiratory viruses, such as respiratory syncytial virus and influenza viruses A and B (Damonte et al. 1994; De Clercq 2000; Bouhlal et al. 2010). Furthermore, the antiviral activity against human immunodeficiency virus (HIV) of red algal extracts has been attributed to the presence of sulphated polysaccharides and lectins, as observed for *Schizymenia pacifica* (Nakashima et al. 1987). Additionally, algal lectins obtained by recombinant production showed a significant activity against hepatitis C virus, as validated in both in vitro and in vivo models (Meuleman et al. 2011; Takebe et al. 2013; Barton et al. 2014).

Overall, the pleiotropic effects of algal extracts as antimicrobial agents may lead to their exploitation as natural ingredients to be incorporated in both food and pharmaceutical preparations for the treatment of multiple infectious diseases. In addition, the widely reported activity against sea and agricultural pathogens open new perspectives in the field of algae valorization for their consideration as natural sources of antibiotics and antivirals.

### Other activities

In addition to the previously described bioactivities associated with red seaweeds, their deriving extracts have been reported in terms of supplementary properties, conferring health-enhancing effects. As a matter of fact, sulphated polysaccharides from *Botryocladia occidentalis* extracts were shown to exert a strong anticoagulant and antithrombotic activity in low doses (Farias et al. 2000; Fonseca et al. 2008). In the same way, sulphated polysaccharides with anticoagulant activities have been also found in other species, such as *Schizymenia binderi, Porphyra haitanensis, Gracilaria debilis*, and *Grateloupia indica* (Sen et al. 1994; Zúñiga et al. 2006; Zhang et al. 2010; Sudharsan et al. 2015). Moreover, the photoprotective effects of pigments from red algae, including carotenoids, together with phenolic compounds, and mycosporine-like amino acids (MAA) have prompted the consideration of derived extracts as efficient additives to be used in already commercialized cosmetic preparations, reporting the effectiveness of such compounds isolated from *Hydropuntia cornea*, *Gracilariopsis longissima*, and *Porphyra umbilicalis* (Álvarez-Gómez et al. 2019). In the case of additional health-promoting properties, red seaweed extracts have been identified as natural sources of neuroprotective agents. Hence, neuroprotection of red algae extracts was reported in terms of acetylcholinesterase (AChE) and butyrylcholinesterase (BuChE), two enzymes closely related with Alzheimer’s and Parkinson’s diseases. For instance, AChE activity has been studied in different compounds extracted from algae which has verified this neuroprotective activity, such as phytol from *Gelidiella acerosa* (tested both in in vitro and in vivo experiments) (Syad et al. 2016) or methanol extracts from *Hypnea valentiae*, *Gracilaria edulis* (Suganthy et al. 2010), *Amphiroa* spp. (Stirk et al. 2007). Finally, the hepatoprotective activity of red algae extracts has been also indicated to be associated with the prevention of oxidative stress. For instance, the oral administration of *Hypnea musciformis* ethanolic extract promoted hepatoprotective activity in liver damage-induced rodent models (Bupesh et al. 2012). Moreover, similar effects were observed by the polyphenol-enriched extracts from *Bryothamnion triquetrum*, (Novoa et al. 2019), the ethanol extract from *Eucheuma cottonii* (Wardani et al. 2017), and the polysaccharide fraction from *Porphyra yezoensis* (Guo et al. 2007).

## Extraction technologies for bioactive compounds

**Fig. 2:**
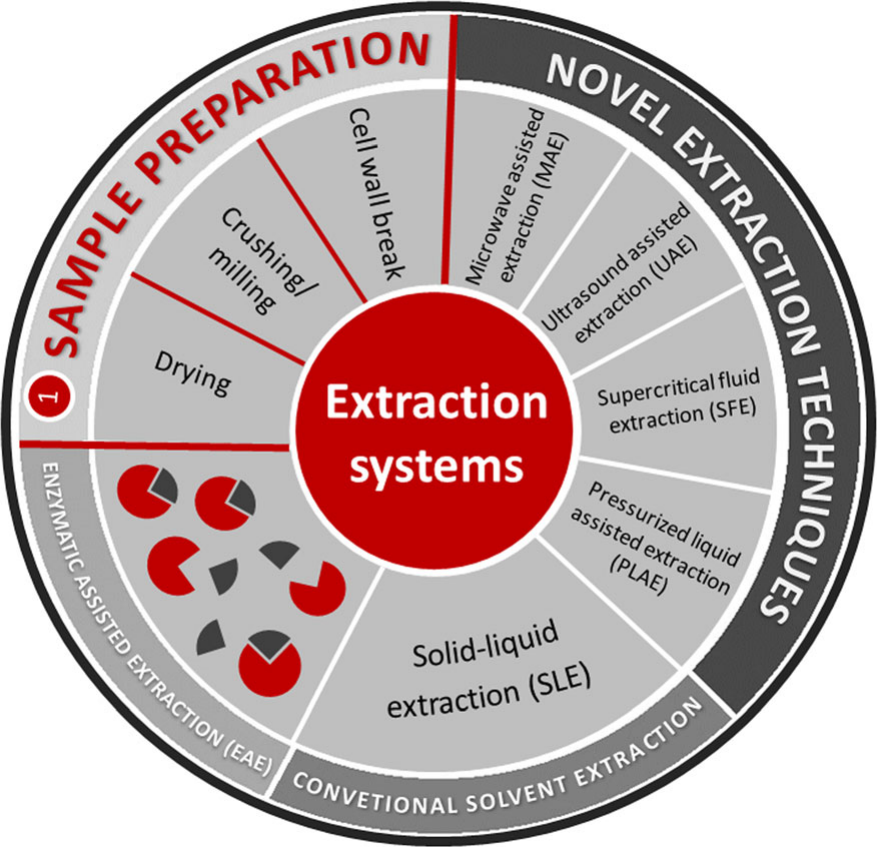
Extraction procedure and different systems for red algae

The bioactive compounds of red seaweeds have been extracted using diverse techniques. Usually, the first step of the extraction process involves a pre-treatment stage with the aim of disrupting algal cell walls and improving the extraction yield (Michalak and Chojnacka 2014). Such pre-treatments can be classified as mechanical, physical, chemical, thermal and enzymatic methods (Fig. 2), and they are highly influenced by the physicochemical nature of target compounds (Michalak and Chojnacka 2014; Jacobsen et al. 2019). A summary of the extraction procedures employed in different studies were included in Table 6.

**Table 6:** Extraction methodologies and conditions for bioactive compounds of red algae

Species	Solvent	Compound	Recovery	Bioactivity	References
Solid/liquid extraction					
*Solieria chordalis*	C/M	Lipids	ns	Antioxidant	Terme et al. (2018)
*Alsidium corallinum*	E/W; M/EA	Chlorophyll a, b and β-carotene	360.38 and 264.24 mg/100 g DW // 93.45 mg/100 g extract	Antioxidant, Antibacterial	Ben Saad et al. (2019)
*Gracilaria* sp.	CC (ionic liquid)	PB	46.5%	ns	Martins et al. (2016)
*Gracilaria gracilis*	Cold water // Hot water // E/W (80:20) // M/W (70:30)	PC	4.76 μg GAE// 5.36 // 3.49 // 4.91	Antioxidant	Heffernan et al. (2014)
Pressurized Liquid Assisted Extraction (PLAE)					
*Hypnea musciformis*	W, 210 °C	PC	39.57 mg GAE/g	Antioxidant	Pangestuti et al. (2019)
*Gracilaria gracilis*	W// E/W (80:20) // M/W (70:30) // 120 °C, 10.34 MPa	PC	2.79 // 2.44 // 3.50 μg GAE	Antioxidant	Heffernan et al. (2014)
*Porphyra tenera*	M/W. 130 °C, 13 MPa	PC	1911 ng/g	Antioxidant	Onofrejová et al. (2010)
*Kappaphycus alvarezii*	W + 1% C4C1im (ionic liquid) 150 °C	Carrageenan	78.75%	Antioxidant	Gereniu et al. (2017)
Supercritical Fluid Extraction (SFE)					
*Solieria chordalis*	CO_2_ + E (8%). 45 °C, 29 MPa	Lipids	ns	Antioxidant	Terme et al. (2018)
*Porphyra* sp., *Hypnea spinella, Chondrus crispus, Halopytis incurvus*	CO_2_ + M/W. 35 MPa, 40 °C	Isoflavones	85.11 // 106.75 // 1114.44// 200.97 ng/g	ns	Klejdus et al. (2010)
Microwave Assisted Extraction (MAE)					
*Porphyridium purpureum*	W, 40 °C	PB	73.7 μg/mg	ns	Juin et al. (2015)
*Solieria chordalis*	W/0,5% KOH, 105 °C	Carrageenan	13.5%	Antiherpetic (HSV-1)	Boulho et al. (2017)
*Gracilaria vermiculophylla*	W/110 °C	Agar	14.8%	ns	Sousa et al. (2010)
*Porphyra haitanensis*	W, 77.84 W	SP	28.98 mL/g	Antitumor	Chen and Xue (2019)
Ultrasound Assisted Extraction (UAE)					
*Porphyra yezoensis*	W, 300 V, 41 °C	Taurine	13.0 mg/g	ns	Wang et al. (2015a)
*Gelidium pusillum*	Phosphate buffer, 30 °C	R-phycoerythrin and R-phycocyanin	0,16 mg/g and 0,11 mg/g	ns	Mittal et al. (2017)
*Osmundea pinnatifida*	W, 50 °C, 400 W	PC and sugars	103.7 μg CE/g and 83 mg/g	Antioxidant	Rodrigues et al. (2015b)
*Gracilaria birdiae*	W + NaOH 0.1 M, 22 °C, 60 W. ED (60 °C, 12 h, pH 8.0)	SP	413 mg	Antioxidant, Anticoagulant	Fidelis et al. (2014)
Enzymatic Assisted Extraction (EAE)					
*Chondracanthus chamissoi*	Cellulase	Proteins	361 mg/g	Antioxidant	Vásquez et al. (2019)
*Palmaria palmata*	Umamizyme	PC	57,1 g GAE/kg	Antioxidant	Wang et al. (2010)
*Chondrus crispus*	Commercial proteases and carbohydrases	ns	40–70% dry matter	Antiviral	Kulshreshtha et al. (2015)
*Osmundea pinnatifida*	Flavourzyme enzymatic complex // Cellulase	PC and sugars	123.1 μg CE/g // 102.2 mg/g	Antioxidant	Rodrigues et al. (2015b)

A (Acetone), W (Water), C (Chloroform), E (Ethanol), M (Methanol), PBS (phosphate buffered saline), DCM (Dichloromethane), EE (Ethyl ether), H (Heptane), EA (Ethyl acetate), PC (Phenolic compounds), SP (Sulphated polysaccharide), ns (not specified), GAE (Gallic Acid Equivalent), CE (Catechol Equivalent), PB (Phycobiliproteins), DW (Dry Weight), ED (Enzymatic Digestions), CC (Cholinium chloride)

### Solid–liquid Extraction

Solid–liquid extraction (SLE) is the simplest and most inexpensive method to extract bioactive compounds, thus being considered as the most widely applied methodology on red seaweed extracts. During SLE protocols, solvent penetrates a pulverized tissue, dissolving the soluble compounds without applying other assisting mechanisms. Maceration or percolation are examples of this type of extraction, in which different organic solvents are used, depending on the solubility of the target compounds. Some of the most used solvents are water, methanol, ethanol, ethyl acetate, either alone or mixed in different proportions (Heffernan et al. 2014; Ben Saad et al. 2019). However, this system presents several disadvantages, such as the high amount of pure solvents, high evaporation rates, low selectivity towards compounds, and long extraction times (Jacobsen et al. 2019). Consequently, SLE also generates high amounts of waste that may lead to a negative environmental impact.

As described in Table 6, numerous studies have used SLE to extract biological compounds from red seaweeds, such as pigments, lipids, phenolic compounds, phycobiliproteins and polysaccharides (Martins et al. 2016; Terme et al. 2018; Ben Saad et al. 2019; Jacobsen et al. 2019).

### Pressurized Liquid Assisted Extraction

Pressurized liquid-assisted exaction (PLE) constitutes an extraction methodology in which solvent preserves the liquid state above its boiling point, by applying high pressure (Michalak and Chojnacka 2014). Different solvents may be used in this extraction, such as water, methanol, and ethanol (Kadam et al. 2015b). Generally, the experimental conditions employed in PLE procedures include a high range of temperatures (120–210 °C), while pressure varies between 10 and 20 MPa (Onofrejová et al. 2010; Heffernan et al. 2014; Pangestuti et al. 2019). This type of extraction is more selective and efficient than SLE and requires significant lower amounts of solvents and shorter extraction times. Nevertheless, due to harsh conditions applied on PLE, this methodology is limited by the thermolabile properties of the compounds subjected to extraction (Kadam et al. 2015a; Jacobsen et al. 2019). Different compounds have been extracted from red algae using this technique, such as phenolic compounds, carbohydrates and proteins (Onofrejová et al. 2010; Heffernan et al. 2014; Gallego et al. 2019; Pangestuti et al. 2019).

### Supercritical Fluid Extraction

Supercritical fluid extraction (SFE), is a novel extraction methodology in which solvents are subjected to high temperatures and pressures to reach a gas–liquid equilibrium, thus improving the extraction yield (Michalak and Chojnacka 2014). The most used solvent in SFE is carbon dioxide (CO_2_) thanks to its availability, low cost, chemical innocuity, and low critical requirements in terms of temperature and pressure conditions (Michalak and Chojnacka 2014; Jacobsen et al. 2019). Pressure values usually range between 29 and 35 MPa, while temperatures varies between 40 and 50 °C, making SFE a suitable technique for the extraction of thermo-labile compounds (Jacobsen et al. 2019). On the other hand, the main drawback of this methodology is the expensive equipment required. Concerning red algae, SFE has been employed to extract specially lipophilic substances, such as glycolipids, phospholipids, and ω-3 fatty acids (Herrero et al. 2006; Klejdus et al. 2010; Michalak and Chojnacka 2014; Terme et al. 2018).

### Microwave Assisted Extraction

Microwave assisted extraction (MAE) is based on the application of electromagnetic radiation with a frequency between 300 MHz and 300 GHz to heat intracellular liquids, which exert pressure on the cell walls and leads to their breakdown. Then, the intracellular compounds are released into the solvent, improving the extraction efficiency (Michalak and Chojnacka 2014). In general, the most used solvent on MAE methodology is water (Sousa et al. 2010; Juin et al. 2015; Boulho et al. 2017; Chen and Xue 2019) and temperature may vary between 40 and 110 °C (Sousa et al. 2010; Juin et al. 2015; Boulho et al. 2017). Therefore, MAE is not recommended to extract temperature-sensitive compounds (Kadam et al. 2015b). Nevertheless, this technique reduces the amount of solvent required and wastes produced, is relatively economic, and easy to perform (Kadam et al. 2015b). Several studies have employed MAE to obtain biological compounds from red algae, such as phycobiliproteins and polysaccharides, including agar and carrageenan (Sousa et al. 2010; Juin et al. 2015; Boulho et al. 2017; Chen and Xue 2019).

### Ultrasound Assisted Extraction

Ultrasound assisted extraction (UAE) is based on the migration of sound waves (whose frequency ranges from 20 to 20,000 Hz), producing micro-bubbles in a liquid medium. These bubbles grow and collapse, disrupting cell walls and, then favoring the penetration of solvents into the matrix (Michalak and Chojnacka 2014; Garcia-Vaquero et al. 2017; Jacobsen et al. 2019). Generally, as it occurs with MAE, water is used as solvent in UAE (Fidelis et al. 2014; Rodrigues et al. 2015b; Wang et al. 2015a). Temperatures usually ranges between 30 and 60 °C, being compatible with the extraction of thermo-labile compounds (Kadam et al. 2015b; Mittal et al. 2017). As a green method, UAE has been reported to improve the extraction yield and reduce the amount of solvent required and the extraction time. In addition, it has high possibilities to be introduced in industrial processes, due to the high scalability to large-scale applications (Garcia-Vaquero et al. 2017). Different studies have employed UAE in the extraction of carbohydrates, sulphated polysaccharides, proteins, amino acids, and phenolic compounds (Fidelis et al. 2014; Rodrigues et al. 2015b; Wang et al. 2015a; Garcia-Vaquero et al. 2017; Mittal et al. 2017).

### Enzymatic Assisted Extraction

Enzymatic assisted extraction (EAE) is a promising system, based on the use of enzymes to hydrolyze the complex and heterogeneous algal cell walls and extract the intracellular compounds. Some examples of the enzymes used are cellulase, α-amilase, pepsin, viscozyme, agarase, etc. (Michalak and Chojnacka 2014; Kadam et al. 2015b; Garcia-Vaquero et al. 2017). The optimal extraction conditions depend on the characteristics of the enzyme, including temperature ranges from 40 to 60 °C and pH from 3.8 to 8 (Michalak and Chojnacka 2014; Garcia-Vaquero et al. 2017). Generally, the extraction is conducted on phosphate or acetate buffer to ensure an efficient enzymatic performance (Praveen et al. 2019; Vásquez et al. 2019). This system presents a high efficiency and specificity, reduced time, and allows reaching great extraction yields (Garcia-Vaquero et al. 2017). In addition, EAE is environmentally friendly and nontoxic, thanks to the independence on pollutant solvents. However, its application at industrial scale is limited, due to the expensive cost of the enzymes (Garcia-Vaquero et al. 2017). Different studies have used EAE to extract biological compounds such as proteins or phenolic compounds (Wang et al. 2010; Rodrigues et al. 2015b; Vásquez et al. 2019).

## Conclusions

Red algae are the largest group of seaweeds and a potential source of bioactive compounds. Among their components, agar and carrageenans account for up to 40–50% of their dry weight and although they are mostly known for their technological and industrial applications as gelling, stabilizing and emulsifying agents, they can be also highlighted as bioactive compounds. The lipid profile of red algae shows a low ω-6/ω-3 rate which indicates beneficial properties in the prevention of cardiovascular diseases. Red algae show high protein content but also lectins have been proposed as cell signaling mediators and antimicrobial compounds. Moreover, minor components such as vitamins (B1, B2, B12, C, β-carotene), minerals (Na, K, Ca, and Mg) and other secondary metabolites (phenolic compounds, terpenoids or alkaloids) are potential candidates to be explored. Concerning the related biological properties, antioxidant, anti-inflammatory, antitumor, and antimicrobial properties can be highlighted as the most studied but more in vivo experiments need to be performed to further disclose the mechanisms of action behind the activities. On the other hand, regarding the extraction techniques, the optimization and development of new procedures using green extraction technologies are necessary to attach to a circular and sustainable economy. Considering these main findings, further work is directed towards the creation and development of new applications to include bioactive compounds from red algae in the food, cosmetic and pharmaceutical industries.

## References

[CR1] Abou Zeid AH, Aboutabl EA, Sleem AA, El-Rafie HM (2014). Water soluble polysaccharides extracted from *Pterocladia capillacea* and *Dictyopteris membranacea* and their biological activities. Carbohydr Polym.

[CR2] Agatonovic-Kustrin S, Morton DW (2017). High-performance thin-layer chromatography HPTLC-direct bioautography as a method of choice for alpha-amylase and antioxidant activity evaluation in marine algae. J Chromatogr A.

[CR3] Ahmed HH, Hegazi MM, Abd-Alla HI (2011). Antitumour and antioxidant activity of some red sea seaweeds in Ehrlich ascites carcinoma in vivo. Zeitschrift fur Naturforsch - Sect C J Biosci.

[CR4] Al-Fadhli A, Wahidulla S, D’Souza L (2006). Glycolipids from the red alga *Chondria armata* (Kütz.) Okamura. Glycobiology.

[CR5] Álvarez-Gómez F, Korbee N, Casas-Arrojo V (2019). UV photoprotection, cytotoxicity and immunology capacity of red algae extracts. Molecules.

[CR6] Antony T, Chakraborty K (2019). First report of antioxidative 2H-chromenyl derivatives from the intertidal red seaweed *Gracilaria salicornia* as potential anti-inflammatory agents. Nat Prod Res.

[CR7] Arulkumar A, Rosemary T, Paramasivam S, Rajendran RB (2018). Phytochemical composition, in vitro antioxidant, antibacterial potential and GC-MS analysis of red seaweeds (*Gracilaria corticata* and *Gracilaria edulis*) from Palk Bay, India. Biocatal Agric Biotechnol.

[CR8] Aziz E, Batool R, Khan MU (2020). An overview on red algae bioactive compounds and their pharmaceutical applications. J Complement Integr Med.

[CR9] Baba M, Snoeck R, Pauwels R, De Clercq E (1988). Sulfated polysaccharides are potent and selective inhibitors of various enveloped viruses, including herpes simplex virus, cytomegalovirus, vesicular stomatitis virus, and human immunodeficiency virus. Antimicrob Agents Chemother.

[CR10] Banskota AH, Stefanova R, Sperker S (2014). Polar lipids from the marine macroalga *Palmaria palmata* inhibit lipopolysaccharide-induced nitric oxide production in RAW264.7 macrophage cells. Phytochemistry.

[CR11] Barbalace MC, Malaguti M, Giusti L (2019). Anti-inflammatory activities of marine algae in neurodegenerative diseases. Int J Mol Sci.

[CR12] Barton C, Kouokam JC, Lasnik AB (2014). Activity of and effect of subcutaneous treatment with the broad- Spectrum antiviral lectin griffithsin in two laboratory rodent models. Antimicrob Agents Chemother.

[CR13] Belghit I, Rasinger JD, Heesch S (2017). In-depth metabolic profiling of marine macroalgae confirms strong biochemical differences between brown, red and green algae. Algal Res.

[CR14] Ben Saad H, Ben Amara I, Kharrat N (2019). Cytoprotective and antioxidant effects of the red alga *Alsidium corallinum* against hydrogen peroxide-induced toxicity in rat cardiomyocytes. Arch Physiol Biochem.

[CR15] Bloch JF, Tardieu-Guigues E (2014). Marine biotechnologies and synthetic biology, new issues for a fair and equitable profit-sharing commercial use. Mar Genom.

[CR16] Bocanegra A, Nieto A, Bastida S (2008). A Nori but not a Konbu, dietary supplement decreases the cholesterolaemia, liver fat infiltration and mineral bioavailability in hypercholesterolaemic growing Wistar rats. Br J Nutr.

[CR17] Boonsri N, Rudtanatip T, Withyachumnarnkul B, Wongprasert K (2017). Protein extract from red seaweed *Gracilaria fisheri* prevents acute hepatopancreatic necrosis disease (AHPND) infection in shrimp. J Appl Phycol.

[CR18] Bouhlal R, Riadi H, Bourgougnon N (2010). Antiviral activity of the extracts of Rhodophyceae from Morocco. African J Biotechnol.

[CR19] Boulho R, Marty C, Freile-Pelegrín Y (2017). Antiherpetic (HSV-1) activity of carrageenans from the red seaweed *Solieria chordalis* (Rhodophyta, Gigartinales) extracted by microwave-assisted extraction (MAE). J Appl Phycol.

[CR20] Bupesh G, Amutha C, Vasanth S (2012). Hepatoprotective efficacy of hypnea muciformis ethanolic extract on CCl4 induced toxicity in rats. Brazil Arch Biol Technol.

[CR21] Campos A, Souza CB, Lhullier C (2012). Anti-tumour effects of elatol, a marine derivative compound obtained from red algae *Laurencia microcladia*. J Pharm Pharmacol.

[CR22] Cardozo KHM, Guaratini T, Barros MP (2007). Metabolites from algae with economical impact. Comp Biochem Physiol - C Toxicol Pharmacol.

[CR23] Castillo S, Heredia N, García S (2015). 2(5H)-Furanone, epigallocatechin gallate, and a citric-based disinfectant disturb quorum-sensing activity and reduce motility and biofilm formation of *Campylobacter jejuni*. Folia Microbiol (praha).

[CR24] Cavallo RA, Acquaviva MI, Stabili L (2013). Antibacterial activity of marine macroalgae against fish pathogenic Vibrio species. Cent Eur J Biol.

[CR25] Chakraborty K, Thilakan B, Raola VK, Joy M (2017). Antibacterial polyketides from *Bacillus amyloliquefaciens* associated with edible red seaweed *Laurenciae papillosa*. Food Chem.

[CR26] Chambers LD, Hellio C, Stokes KR (2011). Investigation of *Chondrus crispus* as a potential source of new antifouling agents. Int Biodeterior Biodegrad.

[CR27] Chan PT, Matanjun P, Yasir SM, Tan TS (2014). Antioxidant and hypolipidaemic properties of red seaweed, *Gracilaria changii*. J Appl Phycol.

[CR28] Chan PT, Matanjun P, Yasir SM, Tan TS (2015). Histopathological studies on liver, kidney and heart of normal and dietary induced hyperlipidaemic rats fed with tropical red seaweed *Gracilaria changii*. J Funct Foods.

[CR29] Chatter R, Ben OR, Rabhi S (2011). *In vivo* and *in vitro* anti-inflammatory activity of neorogioltriol, a new diterpene extracted from the red algae *Laurencia glandulifera*. Mar Drugs.

[CR30] Chaves LDS, Nicolau LAD, Silva RO (2013). Antiinflammatory and antinociceptive effects in mice of a sulfated polysaccharide fraction extracted from the marine red algae *Gracilaria caudata*. Immunopharmacol Immunotoxicol.

[CR31] Chaves RP, da Silva SR, Nascimento Neto LG (2018). Structural characterization of two isolectins from the marine red alga *Solieria filiformis* (Kützing) P.W. Gabrielson and their anticancer effect on MCF-7 breast cancer cells. Int J Biol Macromol.

[CR32] Chen Y-y, Xue Y-t (2019). Optimization of microwave assisted extraction, chemical characterization and antitumor activities of polysaccharides from porphyra haitanensis. Carbohydr Polym.

[CR33] Chen YH, Tu CJ, Wu HT (2004). Growth-inhibitory effects of the red alga *Gelidium amansii* on cultured cells. Biol Pharm Bull.

[CR34] Cho ML, Park GM, Kim SN (2014). Glioblastoma-specific anticancer activity of pheophorbide a from the edible red seaweed *Grateloupia elliptica*. J Microbiol Biotechnol.

[CR35] Cian RE, López-Posadas R, Drago SR (2012). Immunomodulatory properties of the protein fraction from *Phorphyra columbina*. J Agric Food Chem.

[CR36] Cian RE, Drago SR, De Medina FS, Martínez-Augustin O (2015). Proteins and carbohydrates from red seaweeds: evidence for beneficial effects on gut function and microbiota. Mar Drugs.

[CR37] Costa LS, Fidelis GP, Cordeiro SL (2010). Biological activities of sulfated polysaccharides from tropical seaweeds. Biomed Pharmacother.

[CR38] Cotas J, Leandro A, Monteiro P (2020). Seaweed phenolics: from extraction to applications. Mar Drugs.

[CR39] Coura CO, de Araújo IWF, Vanderlei ESO (2012). Antinociceptive and anti-inflammatory activities of sulphated polysaccharides from the red seaweed *Gracilaria cornea*. Basic Clin Pharmacol Toxicol.

[CR40] Cui M, Wu J, Wang S (2019). Characterization and anti-inflammatory effects of sulfated polysaccharide from the red seaweed *Gelidium pacificum* Okamura. Int J Biol Macromol.

[CR41] Cunha L, Grenha A (2016). Sulfated seaweed polysaccharides as multifunctional materials in drug delivery applications. Mar Drugs.

[CR42] Damonte E, Neyts J, Pujol CA (1994). Antiviral activity of a sulphated polysaccharide from the red seaweed *Nothogenia fastigiata*. Biochem Pharmacol.

[CR43] Davies SJ, Soler-vila A, Fitzgerald R, Johnson MP (2019). Macroalgae as a sustainable aquafeed ingredient. Rev Aquacultre.

[CR44] de Almeida CLF, Falcão HdS, Lima GRdM (2011). Bioactivities from marine algae of the genus Gracilaria. Int J Mol Sci.

[CR45] de Gurpilhares DB, Cinelli LP, Simas NK (2019). Marine prebiotics: polysaccharides and oligosaccharides obtained by using microbial enzymes. Food Chem.

[CR46] de Novoa AJV, de Oliveira e Silva AM, Portari Mancini DA (2019). Hepatoprotective properties from the seaweed *Bryothamnion triquetrum* (S.G.Gmelin) M.A.Howe against CCl4-induced oxidative damage in rats. J Pharm Pharmacogn Res.

[CR47] De Clercq E (2000). Current lead natural products for the chemotherapy of human immunodeficiency virus (HIV) infection. Med Res Rev.

[CR48] De Araújo IWF, Vanderlei EDSO, Rodrigues JAG (2011). Effects of a sulfated polysaccharide isolated from the red seaweed *Solieria filiformis* on models of nociception and inflammation. Carbohydr Polym.

[CR49] De Corato U, Salimbeni R, De Pretis A (2017). Antifungal activity of crude extracts from brown and red seaweeds by a supercritical carbon dioxide technique against fruit postharvest fungal diseases. Postharvest Biol Technol.

[CR50] De Inés C, Argandoña VH, Rovirosa J (2004). Cytotoxic activity of halogenated monoterpenes from *Plocamium cartilagineum*. Zeitschrift Fur Naturforsch - Sect C J Biosci.

[CR51] De Sousa Oliveira Vanderlei E, De Araújo IWF, Quinderé ALG (2011). The involvement of the HO-1 pathway in the anti-inflammatory action of a sulfated polysaccharide isolated from the red seaweed *Gracilaria birdiae*. Inflamm Res.

[CR52] Del Val AG, Platas G, Basilio A (2001). Screening of antimicrobial activities in red, green and brown macroalgae from Gran Canaria (Canary Islands, Spain). Int Microbiol.

[CR53] dos Amorim Santos RDN, Rodrigues JAG, Holanda ML (2012). Antimicrobial effect of a crude sulfated polysaccharide from the red seaweed *Gracilaria ornata*. Brazilian Arch Biol Technol.

[CR54] Dworjanyn SA, De Nys R, Steinberg PD (2006). Chemically mediated antifouling in the red alga *Delisea pulchra*. Mar Ecol Prog Ser.

[CR55] El Baz FK, El Baroty GS, Abd El Baky HH (2013). Structural characterization and biological activity of sulfolipids from selected marine algae. Grasas Aceites.

[CR56] Ezzat SM, El Bishbishy MH, Habtemariam S (2018). Looking at marine-derived bioactive molecules as upcoming anti-diabetic agents: a special emphasis on PTP1B inhibitors. Molecules.

[CR57] Farias WRL, Valente AP, Pereira MS, Mourão PAS (2000). Structure and anticoagulant activity of sulfated galactans. Isolation of a unique sulfated galactan from the red algae *Botryocladia occidentalis* and comparison of its anticoagulant action with that of sulfated galactans from invertebrates. J Biol Chem.

[CR58] Fidelis GP, Camara RBG, Queiroz MF (2014). Proteolysis, NaOH and ultrasound-enhanced extraction of anticoagulant and antioxidant sulfated polysaccharides from the edible seaweed, *Gracilaria birdiae*. Molecules.

[CR59] Fleita D, El-Sayed M, Rifaat D (2015). Evaluation of the antioxidant activity of enzymatically-hydrolyzed sulfated polysaccharides extracted from red algae; *Pterocladia capillacea*. LWT - Food Sci Technol.

[CR60] Fonseca RJC, Oliveira SNMCG, Melo FR (2008). Slight differences in sulfation of algal galactans account for differences in their anticoagulant and venous antithrombotic activities. Thromb Haemost.

[CR61] Fuller RW, Cardellina JH, Kato Y (1992). A Pentahalogenated Monoterpene from the red alga *Portieria hornemannii* produces a novel cytotoxicity profile against a diverse panel of human tumor cell lines. J Med Chem.

[CR62] Gallego R, Bueno M, Herrero M (2019). Sub- and supercritical fluid extraction of bioactive compounds from plants, food-by-products, seaweeds and microalgae – an update. TrAC - Trends Anal Chem.

[CR63] García-Pérez P, Barreal ME, Rojo-De Dios L, Rahman A (2019). Bioactive natural products from the genus Kalanchoe as cancer chemopreventive agents: a review. Studies in natural products chemistry.

[CR64] Garcia-Vaquero M, Rajauria G, O’Doherty JV, Sweeney T (2017). Polysaccharides from macroalgae: Recent advances, innovative technologies and challenges in extraction and purification. Food Res Int.

[CR65] Gereniu CRN, Saravana PS, Getachew AT, Chun BS (2017). Characteristics of functional materials recovered from Solomon Islands red seaweed (*Kappaphycus alvarezii*) using pressurized hot water extraction. J Appl Phycol.

[CR66] Gheda S, El-Sheekh M, Abou-Zeid A (2018). *In vitro* anticancer activity of polysaccharide extracted from red alga *Jania rubens* against breast and colon cancer cell lines. Asian Pac J Trop Med.

[CR67] Gómez-Ordóñez E, Jiménez-Escrig A, Rupérez P (2010). Dietary fibre and physicochemical properties of several edible seaweeds from the northwestern Spanish coast. Food Res Int.

[CR68] Gómez-Ordóñez E, Jiménez-Escrig A, Rupérez P (2012). Effect of the red seaweed Mastocarpus stellatus intake on lipid metabolism and antioxidant status in healthy Wistar rats. Food Chem.

[CR69] Gómez-Ordóñez E, Jiménez-Escrig A, Rupérez P (2014). Bioactivity of sulfated polysaccharides from the edible red seaweed <i>Mastocarpus stellatus<(i>. Bioact Carbohydrates Diet Fibre.

[CR70] Guo T-t, Hong-li X, Zhang L-x (2007). In vivo protective effect of *Porphyra yezoensis* polysaccharide against carbon tetrachloride induced hepatotoxicity in mice. Regul Toxicol Pharmacol.

[CR71] Gurgel CFD, Lopez-Bautista J (2007). Red Algae. Encycl Life Sci.

[CR72] Hardouin K, Burlot AS, Umami A (2014). Biochemical and antiviral activities of enzymatic hydrolysates from different invasive French seaweeds. J Appl Phycol.

[CR73] Harnedy PA, FitzGerald RJ (2013). In vitro assessment of the cardioprotective, anti-diabetic and antioxidant potential of *Palmaria palmata* protein hydrolysates. J Appl Phycol.

[CR74] Hayashi K, Walde P, Miyazaki T (2012). Active targeting to osteosarcoma cells and apoptotic cell death induction by the novel Lectin *Eucheuma serra* agglutinin isolated from a marine red alga. J Drug Deliv.

[CR75] He X, Yamauchi A, Nakano T (2019). The composition and anti-inflammatory effect of polysaccharides from the red alga *Chondrus verrucosus*. Fish Sci.

[CR76] Hedberg N, von Schreeb K, Charisiadou S (2018). Habitat preference for seaweed farming – a case study from Zanzibar, Tanzania. Ocean Coast Manag.

[CR77] Heffernan N, Smyth TJ, Fitzgerald RJ (2014). Antioxidant activity and phenolic content of pressurised liquid and solid-liquid extracts from four Irish origin macroalgae. Int J Food Sci Technol.

[CR78] Hellio C, De La Broise D, Dufosse L (2001). Inhibition of marine bacteria by extracts of macroalgae: potential use for environmentally friendly antifouling paints. Mar Environ Res.

[CR79] Herrero M, Cifuentes A, Ibañez E (2006). Sub- and supercritical fluid extraction of functional ingredients from different natural sources: plants, food-by-products, algae and microalgae - a review. Food Chem.

[CR80] Hilliou L, Larotonda FDS, Abreu P (2006). Effect of extraction parameters on the chemical structure and gel properties of κ/ι-hybrid carrageenans obtained from *Mastocarpus stellatus*. Biomol Eng.

[CR81] Holanda ML, Melo VMM, Silva LMCM (2005). Differential activity of a lectin from *Solieria filiformis* against human pathogenic bacteria. Brazilian J Med Biol Res.

[CR82] Holdt SL, Kraan S (2011). Bioactive compounds in seaweed: functional food applications and legislation. J Appl Phycol.

[CR83] Horincar VB, Parfene G, Tyagi AK (2014). Extraction and characterization of volatile compounds and fatty acids from red and green macroalgae from the Romanian Black Sea in order to obtain valuable bioadditives and biopreservatives. J Appl Phycol.

[CR84] Horzum Z, Ozdemir G, Sukatar A, Karabay-Yavasoglu NU (2006). Antimicrobial activity of volatile components and various extracts of the red alga *Jania rubens*. Phyther Res.

[CR85] Iliopoulou D, Mihopoulos N, Vagias C (2003). Novel cytotoxic brominated diterpenes from the red alga *Laurencia obtusa*. J Org Chem.

[CR86] Inoue N, Yamano N, Sakata K (2009). The sulfated polysaccharide porphyran reduces apolipoprotein B100 secretion and lipid synthesis in HepG2 cells. Biosci Biotechnol Biochem.

[CR87] Jacobsen C, Sørensen A-DM, Holdt SL (2019). Source, extraction, characterization, and applications of novel antioxidants from seaweed. Annu Rev Food Sci Technol.

[CR88] Jiang Z, Chen Y, Yao F (2013). Antioxidant, antibacterial and antischistosomal activities of extracts from *Grateloupia livida* (Harv) Yamada. Plos One.

[CR89] Jiao K, Gao J, Zhou T (2019). Isolation and purification of a novel antimicrobial peptide from *Porphyra yezoensis*. J Food Biochem.

[CR90] Juin C, Chérouvrier JR, Thiéry V (2015). Microwave-assisted extraction of phycobiliproteins from *Porphyridium purpureum*. Appl Biochem Biotechnol.

[CR92] Kadam SU, Álvarez C, Tiwari BK, O’Donnell CP (2015a) Extraction of biomolecules from seaweeds. In: Seaweed sustainability: food and non-food applications. Academic Press, pp 243–269. 10.1016/B978-0-12-418697-2.00009-X

[CR91] Kadam SU, Tiwari BK, Smyth TJ, O’Donnell CP (2015). Optimization of ultrasound assisted extraction of bioactive components from brown seaweed *Ascophyllum nodosum* using response surface methodology. Ultrason Sonochem.

[CR93] Karnjana K, Soowannayan C, Wongprasert K (2019). Ethanolic extract of red seaweed *Gracilaria fisheri* and furanone eradicate *Vibrio harveyi* and *Vibrio parahaemolyticus* biofilms and ameliorate the bacterial infection in shrimp. Fish Shellfish Immunol.

[CR94] Kasanah N, Amelia W, Mukminin A (2019). Antibacterial activity of Indonesian red algae *Gracilaria edulis* against bacterial fish pathogens and characterization of active fractions. Nat Prod Res.

[CR95] Kavita K, Singh VK, Jha B (2014). 24-Branched δ5 sterols from *Laurencia papillosa* red seaweed with antibacterial activity against human pathogenic bacteria. Microbiol Res.

[CR96] Kazłowska K, Hsu T, Hou CC (2010). Anti-inflammatory properties of phenolic compounds and crude extract from *Porphyra dentata*. J Ethnopharmacol.

[CR97] Kim KY, Nam KA, Kurihara H, Kim SM (2008). Potent α-glucosidase inhibitors purified from the red alga *Grateloupia elliptica*. Phytochemistry.

[CR98] Kim KY, Nguyen TH, Kurihara H, Kim SM (2010). α-Glucosidase inhibitory activity of bromophenol purified from the red alga *Polyopes lancifolia*. J Food Sci.

[CR99] Klejdus B, Lojková L, Plaza M (2010). Hyphenated technique for the extraction and determination of isoflavones in algae: Ultrasound-assisted supercritical fluid extraction followed by fast chromatography with tandem mass spectrometry. J Chromatogr A.

[CR100] Kubanek J, Prusak AC, Snell TW (2006). Bromophycolides C-I from the Fijian red alga *Callophycus serratus*. J Nat Prod.

[CR101] Kulshreshtha G, Burlot AS, Marty C (2015). Enzyme-assisted extraction of bioactive material from *Chondrus crispus* and *Codium fragile* and its effect on *Herpes simplex* virus (HSV-1). Mar Drugs.

[CR102] Lafarga T, Acién-Fernández FG, Garcia-Vaquero M (2020). Bioactive peptides and carbohydrates from seaweed for food applications: natural occurrence, isolation, purification, and identification. Algal Res.

[CR103] Lalegerie F, Lajili S, Bedoux G (2019). Photo-protective compounds in red macroalgae from Brittany: considerable diversity in mycosporine-like amino acids (MAAs). Mar Environ Res.

[CR104] Lane AL, Stout EP, Lin AS (2009). Antimalarial bromophyeolides J-Q from the Fijian red alga *Callophycus serratus*. J Org Chem.

[CR105] Leal MC, Munro MHG, Blunt JW (2013). Biogeography and biodiscovery hotspots of macroalgal marine natural products. Nat Prod Rep.

[CR106] Liao WR, Lin JY, Shieh WY (2003). Antibiotic activity of lectins from marine algae against marine vibrios. J Ind Microbiol Biotechnol.

[CR107] Lins KOAL, Bezerra DP, Alves APNN (2009). Antitumor properties of a sulfated polysaccharide from the red seaweed *Champia feldmannii* (Diaz-Pifferer). J Appl Toxicol.

[CR108] Liu X, Li X, Gao L (2011). Extraction and PTP1B inhibitory activity of bromophenols from the marine red alga *Symphyocladia latiuscula*. Chinese J Oceanol Limnol.

[CR109] Macartain P, Gill CIR, Brooks M (2007). Special article nutritional value of edible seaweeds. Nutr Rev.

[CR110] Maciel E, Leal MC, Lillebø AI (2016). Bioprospecting of marine macrophytes using MS-based lipidomics as a new approach. Mar Drugs.

[CR111] Makkar F, Chakraborty K (2017). Antidiabetic and anti-inflammatory potential of sulphated polygalactans from red seaweeds *Kappaphycus alvarezii* and *Gracilaria opuntia*. Int J Food Prop.

[CR112] Makkar F, Chakraborty K (2018). Antioxidant and anti-inflammatory oxygenated meroterpenoids from the thalli of red seaweed *Kappaphycus alvarezii*. Med Chem Res.

[CR113] Manefield M, Welch M, Givskov M (2001). Halogenated furanones from the red alga, *Delisea pulchra*, inhibit carbapenem antibiotic synthesis and exoenzyme virulence factor production in the phytopathogen *Erwinia carotovora*. FEMS Microbiol Lett.

[CR114] Manilal A, Sujith S, Selvin J (2010). Antimicrobial potential of marine organisms collected from the southwest coast of India against multiresistant human and shrimp pathogens. Sci Mar.

[CR115] Martins M, Vieira FA, Correia I (2016). Recovery of phycobiliproteins from the red macroalga: *Gracilaria* sp. using ionic liquid aqueous solutions. Green Chem.

[CR116] Matanjun P, Mohamed S, Muhammad K, Mustapha NM (2010). Comparison of cardiovascular protective effects of tropical seaweeds, *Kappaphycus alvarezii*, *Caulerpa lentillifera*, and *Sargassum polycystum*, on high-cholesterol/high-fat diet in rats. J Med Food.

[CR117] Mazumder S, Ghosal PK, Pujol CA (2002). Isolation, chemical investigation and antiviral activity of polysaccharides from *Gracilaria corticata* (Gracilariaceae, Rhodophyta). Int J Biol Macromol.

[CR118] Melo VMM, Medeiros DA, Rios FJB (1997). Antifungal properties of proteins (agglutinins) from the red alga *Hypnea musciformis* (Wulfen) Lamouroux. Bot Mar.

[CR119] Mendes M, Pereira R, Sousa Pinto I (2013). Antimicrobial activity and lipid profile of seaweed extracts from the North Portuguese Coast. Int Food Res J.

[CR120] Meuleman P, Albecka A, Belouzard S (2011). Griffithsin has antiviral activity against hepatitis C virus. Antimicrob Agents Chemother.

[CR121] Michalak I, Chojnacka K (2014). Algal extracts: technology and advances. Eng Life Sci.

[CR122] Mittal R, Tavanandi HA, Mantri VA, Raghavarao KSMS (2017). Ultrasound assisted methods for enhanced extraction of phycobiliproteins from marine macro-algae, *Gelidium pusillum* (*Rhodophyta*). Ultrason Sonochem.

[CR123] Mohamed S, Hashim SN, Rahman HA (2012). Seaweeds: a sustainable functional food for complementary and alternative therapy. Trends Food Sci Technol.

[CR124] Mohy El-Din SM, El-Ahwany AMD (2016). Bioactivity and phytochemical constituents of marine red seaweeds (*Jania rubens, Corallina mediterranea *and P*terocladia capillacea*). J Taibah Univ Sci.

[CR125] Moreira AS, González-Torres L, Olivero-David R (2010). Wakame and Nori in restructured meats included in cholesterol-enriched diets affect the antioxidant enzyme gene expressions and activities in Wistar rats. Plant Foods Hum Nutr.

[CR126] Nakashima H, Kido Y, Kobayashi N (1987). Purification and characterization of an avian myeloblastosis and human immunodeficiency virus reverse transcriptase inhibitor, sulfated polysaccharides extracted from sea algae. Antimicrob Agents Chemother.

[CR127] Namvar F, Mohamed S, Fard SG (2012). Polyphenol-rich seaweed (*Eucheuma cottonii*) extract suppresses breast tumour via hormone modulation and apoptosis induction. Food Chem.

[CR128] Necas J, Bartosikova L (2013). Carrageenan: a review. Vet Med (praha).

[CR129] Niu J-F, Wang G-C, Zhou B-C (2007). Purification of R-phycoerythrin from *Porphyra haitanensis* (Bangiales, Rhodophyta) using expanded-bed absorption. J Phycol.

[CR130] Onofrejová L, Vašíčková J, Klejdus B (2010). Bioactive phenols in algae: the application of pressurized-liquid and solid-phase extraction techniques. J Pharm Biomed Anal.

[CR131] Osman MEH, Abushady AM, Elshobary ME (2010). In vitro screening of antimicrobial activity of extracts of some macroalgae collected from Abu-Qir bay Alexandria. Egypt African J Biotechnol.

[CR132] Øverland M, Mydland LT, Skrede A (2019). Marine macroalgae as sources of protein and bioactive compounds in feed for monogastric animals. J Sci Food Agric.

[CR133] Palermo JA, Flower PB, Seldes AM (1992). Chondriamides A and B, new indolic metabolites from the red alga *Chondria* sp. Tetrahedron Lett.

[CR134] Pangestuti R, Kim SK (2014). Biological activities of Carrageenan.

[CR135] Pangestuti R, Getachew AT, Siahaan EA, Chun BS (2019). Characterization of functional materials derived from tropical red seaweed *Hypnea musciformis* produced by subcritical water extraction systems. J Appl Phycol.

[CR136] Panlasigui LN, Baello OQ, Dimatangal JM, Dumelod BD (2003). Blood cholesterol and lipid-lowering effects of carrageenan on human volunteers. Asia Pac J Clin Nutr.

[CR137] Patil NP, Le V, Sligar AD (2018). Algal polysaccharides as therapeutic agents for atherosclerosis. Front Cardiovasc Med.

[CR138] Pereira L (2011) A review of the nutrient composition of selected edible seawedds. In: Seaweed: Ecology, nutrient composition and medicinal uses. Nova Science Publishers, Inc, pp 242–254.

[CR139] Philippus AC, Zatelli GA, Wanke T (2018). Molecular networking prospection and characterization of terpenoids and C15-acetogenins in Brazilian seaweed extracts. RSC Adv.

[CR140] Prado-Fernández J, Rodríguez-Vázquez JA, Tojo E, Andrade JM (2003). Quantitation of κ-, ι- and λ-carrageenans by mid-infrared spectroscopy and PLS regression. Anal Chim Acta.

[CR141] Praveen MA, Parvathy KRK, Balasubramanian P, Jayabalan R (2019). An overview of extraction and purification techniques of seaweed dietary fibers for immunomodulation on gut microbiota. Trends Food Sci Technol.

[CR142] Ren D, Bedzyk LA, Ye RW (2004). Differential gene expression shows natural brominated furanones interfere with the autoinducer-2 bacterial signaling system of Escherichia coli. Biotechnol Bioeng.

[CR143] Rhimou B, Hassane R, José M, Nathalie B (2010). The antibacterial potential of the seaweeds (Rhodophyceae) of the Strait of Gibraltar and the Mediterranean coast of Morocco. African J Biotechnol.

[CR144] Rocha De Souza MC, Marques CT, Guerra Dore CM (2007). Antioxidant activities of sulfated polysaccharides from brown and red seaweeds. J Appl Phycol.

[CR145] Rodrigues D, Alves C, Horta A (2015). Antitumor and antimicrobial potential of bromoditerpenes isolated from the Red Alga, *Sphaerococcus coronopifolius*. Mar Drugs.

[CR146] Rodrigues D, Freitas AC, Pereira L (2015). Chemical composition of red, brown and green macroalgae from Buarcos bay in Central West Coast of Portugal. Food Chem.

[CR147] Rodrigues D, Sousa S, Silva A (2015). Impact of enzyme- and ultrasound-assisted extraction methods on biological properties of red, brown, and green seaweeds from the Central West Coast of Portugal. J Agric Food Chem.

[CR148] Rosemary T, Arulkumar A, Paramasivam S (2019). Biochemical, micronutrient and physicochemical properties of the dried red seaweeds gracilaria edulis and gracilaria corticata. Molecules.

[CR149] Rudtanatip T, Lynch SA, Wongprasert K, Culloty SC (2018). Assessment of the effects of sulfated polysaccharides extracted from the red seaweed Irish moss *Chondrus crispus* on the immune-stimulant activity in mussels *Mytilus* spp. Fish Shellfish Immunol.

[CR150] Rupérez P (2002). Mineral content of edible marine seaweeds. Food Chem.

[CR151] Ruqqia K, Sultana V, Ara J (2014). Hypolipidaemic potential of seaweeds in normal, triton-induced and high-fat diet-induced hyperlipidaemic rats. J Appl Phycol.

[CR152] Salta M, Wharton JA, Dennington SP (2013). Anti-biofilm performance of three natural products against initial bacterial attachment. Int J Mol Sci.

[CR153] Sangha JS, Fan D, Banskota AH (2013). Bioactive components of the edible strain of red alga, Chondrus crispus, enhance oxidative stress tolerance in *Caenorhabditis elegans*. J Funct Foods.

[CR154] Sen AK, Das AK, Banerji N (1994). A new sulfated polysaccharide with potent blood anti-coagulant activity from the red seaweed *Grateloupia indica*. Int J Biol Macromol.

[CR155] Shao P, Chen X, Sun P (2013). In vitro antioxidant and antitumor activities of different sulfated polysaccharides isolated from three algae. Int J Biol Macromol.

[CR156] Shi D, Li J, Jiang B (2012). Bromophenols as inhibitors of protein tyrosine phosphatase 1B with antidiabetic properties. Bioorg Med Chem Lett.

[CR157] Shoeib NA, Bibby MC, Blunden G (2004). In-vitro cytotoxic activities of the major bromophenols of the red alga *Polysiphonia lanosa* and some novel synthetic isomers. J Nat Prod.

[CR158] Silva TH, Alves A, Popa EG (2012). Marine algae sulfated polysaccharides for tissue engineering and drug delivery approaches. Biomatter.

[CR159] Singh RS, Walia AK (2018). Lectins from red algae and their biomedical potential. J Appl Phycol.

[CR160] Škrovánková S, Kim SK (2011). Seaweed vitamins as nutraceuticals. Advances in food and nutrition research.

[CR161] Smit AJ (2004). Medicinal and pharmaceutical uses of seaweed natural products: a review. J Appl Phycol.

[CR162] Smyrniotopoulos V, Vagias C, Rahman MM (2008). Brominated Diterpenes with antibacterial activity from the red alga *Sphaerococcus coronopifolius*. J Nat Prod.

[CR163] Smyrniotopoulos V, Vagias C, Bruyère C (2010). Structure and in vitro antitumor activity evaluation of brominated diterpenes from the red alga *Sphaerococcus coronopifolius*. Bioorganic Med Chem.

[CR164] Soares AR, Robaina MCS, Mendes GS (2012). Antiviral activity of extracts from Brazilian seaweeds against herpes simplex virus. Brazilian J Pharmacogn.

[CR165] Sousa AMM, Alves VD, Morais S (2010). Agar extraction from integrated multitrophic aquacultured *Gracilaria vermiculophylla*: evaluation of a microwave-assisted process using response surface methodology. Bioresour Technol.

[CR166] Souza RB, Frota AF, Silva J (2018). *In vitro* activities of kappa-carrageenan isolated from red marine alga *Hypnea musciformis*: antimicrobial, anticancer and neuroprotective potential. Int J Biol Macromol.

[CR167] Souza CRM, Bezerra WP, Souto JT (2020). Marine alkaloids with anti-inflammatory activity: current knowledge and future perspectives. Mar Drugs.

[CR168] Stabili L, Acquaviva MI, Biandolino F (2012). The lipidic extract of the seaweed *Gracilariopsis longissima* (Rhodophyta, Gracilariales): A potential resource for biotechnological purposes?. N Biotechnol.

[CR169] Stirk WA, Reinecke DL, Van SJ (2007). Seasonal variation in antifungal, antibacterial and acetylcholinesterase activity in seven South African seaweeds. J Appl Phycol.

[CR170] Sudhakar K, Mamat R, Samykano M (2018). An overview of marine macroalgae as bioresource. Renew Sustain Energy Rev.

[CR171] Sudharsan S, Subhapradha N, Seedevi P (2015). Antioxidant and anticoagulant activity of sulfated polysaccharide from *Gracilaria debilis* (Forsskal). Int J Biol Macromol.

[CR172] Sugahara T, Ohama Y, Fukuda A (2001). The cytotoxic effect of *Eucheuma serra* agglutinin (ESA) on cancer cells and its application to molecular probe for drug delivery system using lipid vesicles. Cytotechnology.

[CR173] Suganthy N, Karutha Pandian S, Pandima Devi K (2010). Neuroprotective effect of seaweeds inhabiting South Indian coastal area (Hare Island, Gulf of Mannar Marine Biosphere Reserve): cholinesterase inhibitory effect of *Hypnea valentiae* and *Ulva reticulata*. Neurosci Lett.

[CR174] Suleria HAR, Gobe G, Masci P, Osborne SA (2016). Marine bioactive compounds and health promoting perspectives; Innovation pathways for drug discovery. Trends Food Sci Technol.

[CR175] Suwal S, Perreault V, Marciniak A (2019). Effects of high hydrostatic pressure and polysaccharidases on the extraction of antioxidant compounds from red macroalgae, *Palmaria palmata* and *Solieria chordalis*. J Food Eng.

[CR176] Syad AN, Rajamohamed BS, Shunmugaiah KP, Kasi PD (2016). Neuroprotective effect of the marine macroalga *Gelidiella acerosa*: identification of active compounds through bioactivity-guided fractionation. Pharm Biol.

[CR177] Takebe Y, Saucedo CJ, Lund G (2013). Antiviral lectins from red and blue-green algae show potent in vitro and in vivo activity against hepatitis C virus. PLoS ONE.

[CR178] Terme N, Boulho R, Kucma JP (2018). Radical scavenging activity of lipids from seaweeds isolated by solid-liquid extraction and supercritical uids. OCL.

[CR179] Thompson CC, Kruger RH, Thompson FL (2017). Unlocking marine biotechnology in the developing world. Trends Biotechnol.

[CR180] Torres MD, Flórez-Fernández N, Domínguez H (2019). Integral utilization of red seaweed for bioactive production. Mar Drugs.

[CR181] Tsuge K, Okabe M, Yoshimura T (2004). Dietary effects of porphyran from *Porphyra yezoensis* on growth and lipid metabolism of Sprague-Dawley rats. Food Sci Technol Res.

[CR182] Vairappan CS (2003). Potent antibacterial activity of halogenated metabolites from Malaysian red algae, *Laurencia majuscula* (Rhodomelaceae, Ceramiales). Biomol Eng.

[CR183] Vairappan CS, Daitoh M, Suzuki M (2001). Antibacterial halogenated metabolites from the Malaysian *Laurencia* species. Phytochemistry.

[CR184] Valderrama D, Cai J, Hishamunda N, Ridler N (2013) Social and economic dimensions of carrageenan seaweed farming

[CR185] Vásquez V, Martínez R, Bernal C (2019). Enzyme-assisted extraction of proteins from the seaweeds *Macrocystis pyrifera* and *Chondracanthus chamissoi*: characterization of the extracts and their bioactive potential. J Appl Phycol.

[CR186] Villanueva MJ, Morcillo M, Tenorio MD (2014). Health-promoting effects in the gut and influence on lipid metabolism of *Himanthalia elongata* and *Gigartina pistillata* in hypercholesterolaemic Wistar rats. Eur Food Res Technol.

[CR187] Wang W, Okada Y, Shi H (2005). Structures and aldose reductase inhibitory effects of bromophenols from the red alga *Symphyocladia latiuscula*. J Nat Prod.

[CR188] Wang T, Jónsdóttir R, Kristinsson HG (2010). Enzyme-enhanced extraction of antioxidant ingredients from red algae *Palmaria palmata*. LWT - Food Sci Technol.

[CR189] Wang F, Guo XY, Zhang DN (2015). Ultrasound-assisted extraction and purification of taurine from the red algae *Porphyra yezoensis*. Ultrason Sonochem.

[CR190] Wang LJ, Jiang B, Wu N (2015). Natural and semisynthetic protein tyrosine phosphatase 1B (PTP1B) inhibitors as anti-diabetic agents. RSC Adv.

[CR191] Wang X, Li W, Xiao L (2017). In vivo antihyperlipidemic and antioxidant activity of porphyran in hyperlipidemic mice. Carbohydr Polym.

[CR192] Wardani G, Farida N, Andayani R (2017). The potency of red seaweed (*Eucheuma cottonii*) extracts as hepatoprotector on lead acetate-induced hepatotoxicity in mice. Pharmacognosy Res.

[CR193] Wen X, Peng C, Zhou H (2006). Nutritional composition and assessment of *Gracilaria lemaneiformis* Bory. J Integr Plant Biol.

[CR194] Wijesinghe WAJP, Kang MC, Lee WW (2014). 5β-Hydroxypalisadin B isolated from red alga *Laurencia snackeyi* attenuates inflammatory response in lipopolysaccharide-stimulated RAW 264.7 macrophages. Algae.

[CR195] World Health Organization (2019) World health statistics 2019: monitoring health for the SDGs, sustainable development goals. Geneva

[CR196] Xu F, Wang F, Wang Z (2016). Glucose uptake activities of bis (2, 3-dibromo-4, 5-dihydroxybenzyl) ether, a novel marine natural product from red alga *Odonthalia corymbifera* with protein tyrosine phosphatase 1b inhibition, *in vitro* and *in vivo*. PLoS One.

[CR197] Yuan YV, Carrington MF, Walsh NA (2005). Extracts from dulse (*Palmaria palmata*) are effective antioxidants and inhibitors of cell proliferation in vitro. Food Chem Toxicol.

[CR198] Zhang Z, Zhang Q, Wang J (2010). Regioselective syntheses of sulfated porphyrans from *Porphyra haitanensis* and their antioxidant and anticoagulant activities *in vitro*. Carbohydr Polym.

[CR199] Zhou G, Xin H, Sheng W (2005). *In vivo* growth-inhibition of S180 tumor by mixture of 5-Fu and low molecular λ-carrageenan from *Chondrus ocellatus*. Pharmacol Res.

[CR200] Zhou G, Sheng W, Yao W, Wang C (2006). Effect of low molecular λ-carrageenan from *Chondrus ocellatus* on antitumor H-22 activity of 5-Fu. Pharmacol Res.

[CR201] Zhou C, Yu X, Zhang Y (2012). Ultrasonic degradation, purification and analysis of structure and antioxidant activity of polysaccharide from *Porphyra yezoensis* Udea. Carbohydr Polym.

[CR202] Zúñiga EA, Matsuhiro B, Mejías E (2006). Preparation of a low-molecular weight fraction by free radical depolymerization of the sulfated galactan from *Schizymenia binderi* (Gigartinales, Rhodophyta) and its anticoagulant activity. Carbohydr Polym.

